# Hydrazides as Powerful Tools in Medicinal Chemistry: Synthesis, Reactivity, and Biological Applications

**DOI:** 10.3390/molecules30132852

**Published:** 2025-07-03

**Authors:** Sofia Teixeira, Elisabete M. S. Castanheira, M. Alice Carvalho

**Affiliations:** 1 Centre of Chemistry of University of Minho (CQ-UM), Campus de Gualtar, 4710-057 Braga, Portugal; id9191@alunos.uminho.pt; 2 Centre of Physics of Minho and Porto Universities (CF-UM-UP), University of Minho, Campus de Gualtar, 4710-057 Braga, Portugal; ecoutinho@fisica.uminho.pt

**Keywords:** hydrazides, hydrazide derivatives, synthesis, bioactive hydrazide derivatives

## Abstract

The increase in drug resistance and the high toxicity of current drugs have inspired the scientific community to develop new drugs for various diseases. Hydrazides have become an attractive functional group to easily obtain a plethora of novel compounds with a broad range of biological activities. This review, which contains studies in the literature from the previous five years, focuses on the synthesis methods and biological applications of hydrazides and their derivatives. Here, the details of the experimental reaction conditions used for the synthesis of hydrazides and their derivatives (hydrazide–hydrazones and heterocycle derivatives) are presented, as well as the purification methods and the biological activity of the synthesized compounds.

## 1. Introduction

According to the World Health Organization [1], diabetes, cardiovascular (ischemic heart disease and stroke), respiratory (chronic obstructive pulmonary disease and respiratory infections), cancers, and Alzheimer’s and dementia diseases are some of the leading causes of death worldwide. Besides these diseases, malaria, tuberculosis, HIV/AIDS, and cirrhosis of the liver are among the leading causes of death in low-income countries. The main obstacles to treating/eradicating these diseases include the increase in drug resistance to current drugs [2,3,4,5,6,7,8,9] and the high toxicity of the drugs used in the treatments [2,10,11,12].

Nowadays, many efforts have been made to develop novel and safer therapeutic alternatives. The scientific community has been searching for new compounds with reduced toxicity and improved biological efficacy, or even potential probes for bioimaging in disease diagnosis [13].

Hydrazides are a class of organic compounds with the functional group R-CON-R^1^N-R^2^R^3^ [14]. It is an extremely important group in organic chemistry, being an effective substrate in both domains of chemical reactions and medicinal chemistry [15,16].

**Figure 1:**
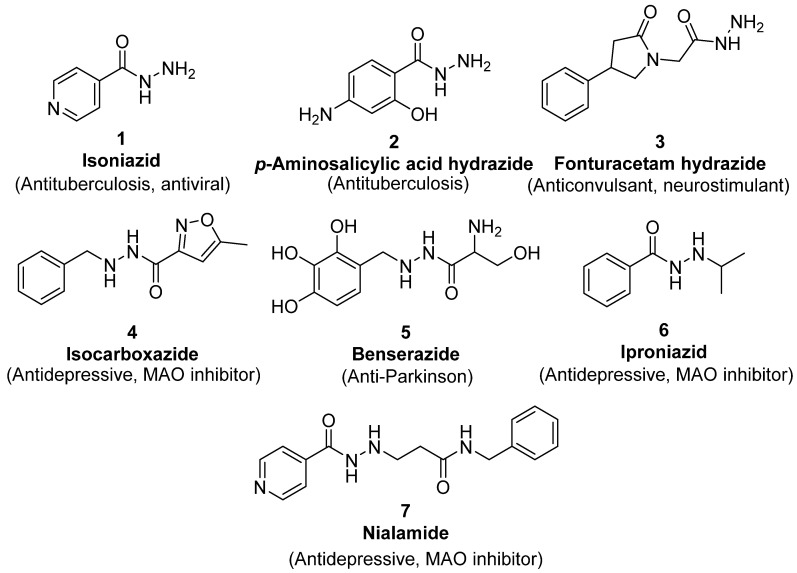
Examples of hydrazides and their therapeutic applications.

Since the 20th century, several hydrazides such as isoniazid **1** (isonicotinic acid hydrazide) [9,17,18], *p*-aminosalicylic acid hydrazide **2** [19], fonturacetam hydrazide **3** [20,21], isocarboxazide **4** [22], iproniazid **6** [23], nialamide **7** [11] and benserazide **5** [24] have been introduced for therapeutic purposes, as antituberculosis, antiviral, anticonvulsant, neurostimulator, antidepressive (monoamine oxidase inhibitor), and anti-Parkinson agents (Figure 1).

**Figure 2:**
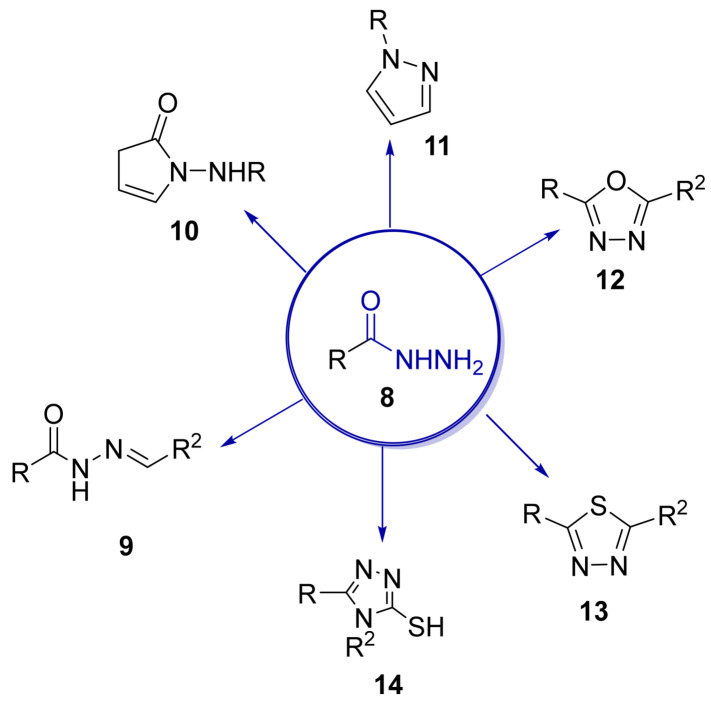
Linear and heterocyclic compounds synthesized from hydrazides.

Over time, hydrazides **8** were found to be great precursors of other bioactive compounds such as hydrazine–hydrazones **9** [25,26]. Hydrazides **8** were also employed as building block synthons of different classes of heterocycles, like pyrrolones **10** [27,28], pyrazoles **11** [29,30], oxadiazoles **12** [31], thiadiazoles **13** [30,32], triazoles **14** [30], by cyclization or cycloaddition reactions with other reagents [28,33] (Figure 2). These hydrazide derivatives similarly revealed a wide range of biological activities, including antitumor [34,35,36,37], antimicrobial [38], antifungal [39], antimalarial [2], antileishmanial [40], anti-inflammatory [41], antidiabetic [42,43] and antioxidant [44] properties. They also showed herbicide activity or were used as dyes [45,46].

A review describing the synthesis of hydrazides and heterocyclic rings from hydrazides was published in 2014 by Majumdar et al. [47]. In 2018, Hosseini et al. [32] reported a compilation of the synthesis of heterocycles from cyanoacetohydrazides. Also, in 2021, Mali et al. [48] briefly reported the importance of the hydrazides and their derivatives (specifically hydrazide–hydrazones) as bioactive compounds over the years.

This review presents a comprehensive compilation and description of the methods used to synthesize hydrazides in the last 5 years, their use as precursors or synthons to generate new derivatives, and their main biological applications.

Moreover, throughout the review, tables will be provided summarizing the experimental conditions for the synthesis and purification of hydrazides and their derivatives.

## 2. Hydrazides

### 2.1. Synthesis of Hydrazides

**Scheme 1:**
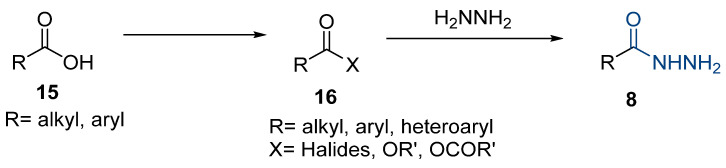
Representative scheme of hydrazide synthesis.

The first existing hydrazides, specifically formic and acetic acid hydrazides, were produced by Kurzius in 1895 [49]. Currently, many hydrazides with alkyl, aryl, and heteroaryl substituents are being synthesized to overcome drug resistance and toxicity. Hydrazides **8** (Scheme 1) are conventionally synthesized from compounds **16**, such as esters [2,13,26,34,35,36,37,38,39,40,41,43,44,50,51,52,53,54,55,56,57,58,59,60,61,62,63,64,65,66,67,68,69,70,71,72,73,74,75,76,77,78,79,80,81,82,83,84,85,86,87,88,89,90,91,92,93,94,95], anhydrides [96], and acyl chlorides [2,97,98], or others [99,100,101,102,103] that possess a good leaving group, and hydrazine. When it is not possible to have compounds **16** available, the leaving group is produced from the acid derivative **15** [82,85,88,96]. According to the reaction conditions outlined in Table 1, the experimental reaction conditions to generate hydrazide **8** from precursor **16** do not differ much, and usually, the reaction takes place in an alcohol solvent, at room temperature, or under reflux. The reactions did not last more than 24 h, and generally, product **8** was purified by recrystallization or by column chromatography. Hydrazides **8a** to **8abb** were obtained in low to excellent yields (26–98%) from esters (**17a**–**17aaw**), anhydrides (**18**), acyl chlorides (**19a**–**c**), or others (**20**–**23**).

**Scheme 2:**
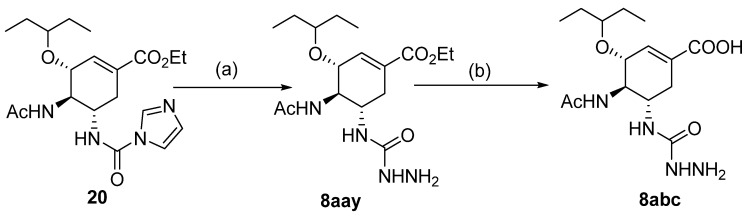
Synthesis of hydrazide **8abc**. (a) H_2_NNH_2_·H_2_O, Et_3_N, Na_2_SO_4_, CHCl_3_, 35 °C; (b) NaOH, MeOH/H_2_O, r.t.

Besides the regular functional groups, Zhao et al. [99] obtained the hydrazide **8aay** from activated intermediary **20** by reaction with hydrazine hydrate (Scheme 2). The acid derivative **8abc** was obtained from **8aay** by reaction with sodium hydroxide in aqueous methanol.

**Scheme 3:**
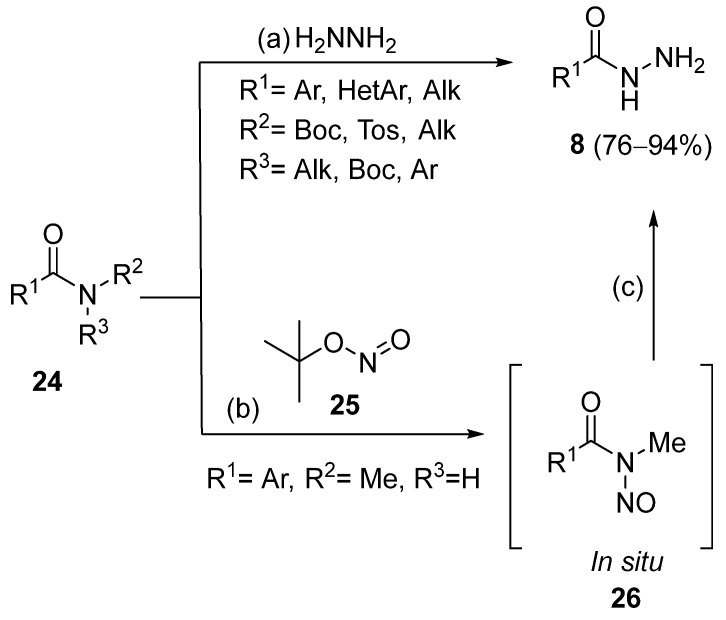
Synthesis of hydrazides 8 by transamidation; (a) _2_HNNH_2_ (2-3 eq), DCM, r.t., 30 min–2 h; when R^2^ = Boc and R^3^ = Me, or R^1^ = Alk, DBU (1 eq) is also used; (b) **25** (3 eq), DCM, r.t., 1 h; (c) _2_HNNH_2_ (3 eq), DCM, r.t., 1 h.

Singh et al. [104] obtained hydrazides via the transamidation of *N*-Boc, *N*-nitroso, and *N*-tosyl amides with hydrazine hydrate, at room temperature, in 76–94% yields (Scheme 3).

**Table 1:** Reaction conditions for the synthesis and purification of hydrazides from esters, anhydrides, acyl chlorides, and others.

Ref.	Starting Material	Experimental Conditions	Purification Process	Hydrazide Compounds (η%)
[50]	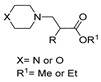 **17a**	Hydrazine hydrate (1.2 eq) EtOH 75–80 °C 2 h	Silica gel column chromatography	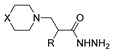 **8a** X = N, O; R = Alk (70–95%)
[51]	*** 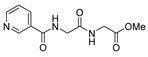 *** **17b**	Hydrazine hydrate (10 eq) MeOH Reflux 3 h	-	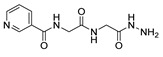 **8b**
[26]	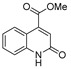 **17c**	Hydrazine hydrate (n.s.) EtOH or MeOH 85 °C 6 h	Recrystallization from aqueous ethanol or methanol	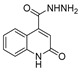 **8c** (88%)
[26]	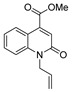 **17d**	Hydrazine hydrate (n.s.) EtOH or MeOH 85 °C 6 h	Recrystallization from aqueous ethanol or methanol	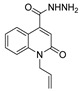 **8d** (54%)
[26]	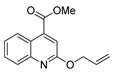 **17e**	Hydrazine hydrate (n.s.) EtOH or MeOH 85 °C 6 h	Recrystallization from aqueous ethanol or methanol	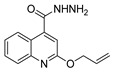 **8e** (75%)
[26]	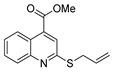 **17f**	Hydrazine hydrate (n.s.) EtOH or MeOH 85 °C 6 h	Recrystallization from aqueous ethanol or methanol	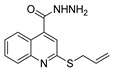 **8f** (83%)
[55]	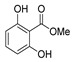 **17g**	Hydrazine hydrate (10 eq added dropwise) MeOH Reflux 2 h	-	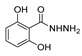 **8g** (80%)
[56]	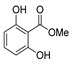 **17g**	Hydrazine hydrate (9 eq added dropwise) MeOH Reflux 2 h	Silica gel column chromatography	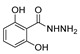 **8g** (55%)
[57]	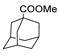 **17h**	Hydrazine hydrate (80%) (20.6 eq) Neat Reflux n.s.	-	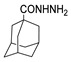 **8h** (96%)
[43]	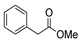 **17i**	Hydrazine 80% (n.s.) EtOH Reflux 6h	n.s.	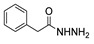 **8i** (50%)
[43]	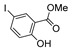 **17j**	Hydrazine 80% (n.s.) EtOH Reflux 6 h	n.s.	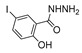 **8j** (57%)
[43]	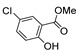 **17k**	Hydrazine 80% (n.s.) EtOH Reflux 6 h	n.s.	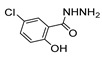 **8k** (47%)
[13]	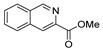 **17l**	Hydrazine hydrate (1.7 eq) MeOH Reflux 4.5 h	Recrystallization from methanol	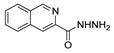 **8l** (63%)
[34]	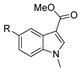 **17m**	Hydrazine hydrate (n.s.) EtOH Reflux 8–10 h	n.s.	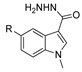 **8m** R = H, Br (n.s.)
[38]	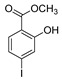 **17n**	Hydrazine hydrate (1.1 eq) EtOH Reflux 3 h	Recrystallization from ethanol	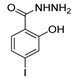 **8n** (75%)
[40]	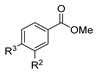 **17o** R^2^ = H, R^3^ = H **17p** R^2^ = Cl, R^3^ = H **17q** R^2^ = H, R^3^ = Cl	Hydrazine hydrate 80% (n.s.) EtOH Reflux 3 h	n.s.	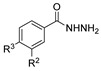 **8o** R^2^ = H, R^3^ = H **8p** R^2^ = Cl, R^3^ = H **8q** R^2^ = H, R^3^ = Cl (n.s.)
[53]	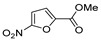 **17r**	Hydrazine hydrate (1 eq) Anhydrous EtOH ~0 °C 30 min	Recrystallization from ethanol	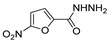 **8r** (73%)
[54]	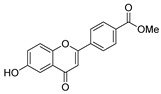 **17s**	Hydrazine hydrate (n.s.) MeOH Reflux 6 h	Recrystallization from ethanol	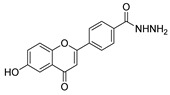 **8s** (n.s.)
[39]	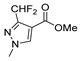 **17t**	Hydrazine monohydrate (8 eq) EtOH Ice bath 30 min	-	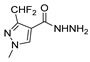 **8t** (n.s.)
[59]	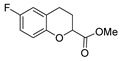 **17u**	Hydrazine hydrate (2 eq) EtOH Reflux 8 h	-	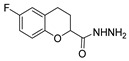 **8u** (91%)
[105]	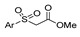 **17v** 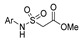 **17w**	Hydrazine hydrate (1.1 eq) MeOH Pyridine (cat.) Reflux 6–7 h	Recrystallization from methanol	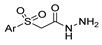 **8v** 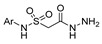 **8w** (n.s)
[61]	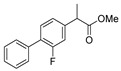 **17x**	Hydrazine hydrate (2 eq) EtOH Reflux 4 h	-	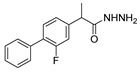 **8x** (n.s.)
[62]	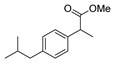 **17y**	Hydrazine hydrate 80% (~8 eq, dropwise) EtOH 95–100 °C 12 h	Recrystallization from ethanol	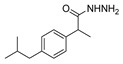 **8y** (89%)
[36]	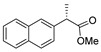 **17z**	Hydrazine hydrate (n.s.) EtOH 80–90 °C 8 h	-	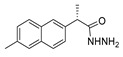 **8z** (n.s.)
[41]	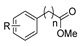 **17aa**	Hydrazine hydrate 85% (~6.5 eq) MeOH Reflux 8 h	-	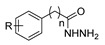 **8aa** R = Me, OMe (82–92%)
[63]	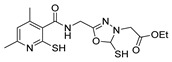 **17ab**	Hydrazine hydrate (~10 eq) MeOH Reflux 5 h	Recrystallization from methanol	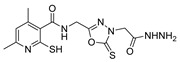 **8ab** (37%)
[64]	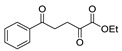 **17ac**	Hydrazine hydrate (n.s.) EtOH Reflux 6 h	-	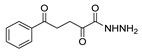 **8ac** (78%)
[35]	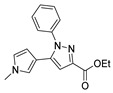 **17ad**	Hydrazine (4 eq) EtOH Reflux 5 h	Recrystallization from ethanol	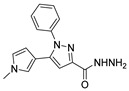 **8ad** (73%)
[65]	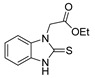 **17ae**	Hydrazine hydrate (6 eq.) EtOH 30 °C 1 h	Recrystallization from ethanol	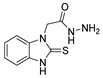 **8ae** (26%)
[66]	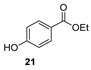 **17af**	Hydrazine hydrate 80% (31 eq) EtOH Reflux 8 h	Recrystallization from ethanol	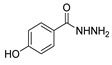 **8af** (n.s.)
[67]	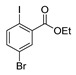 **17ag**	Hydrazide hydrate (4.6 eq) EtOH Reflux 3 h	Recrystallization from ethanol	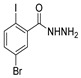 **8ag** (78%)
[68]	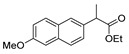 **17ah**	Hydrazine hydrate 80% (31 eq) EtOH Reflux 4 h	Recrystallization from ethanol	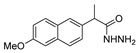 **8ah** (70%)
[43]	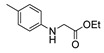 **17ai**	Hydrazine hydrate 80% (15 eq) EtOH Reflux 8 h	Recrystallization from ethanol	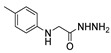 **8ai** (63%)
[43]	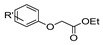 **17aj**	Hydrazine 80% (n.s.) EtOH Reflux 6 h	n.s.	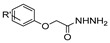 **8aj** R′ = Alk, Halide (39–54%)
[43]	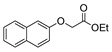 **17ak**	Hydrazine 80% (n.s.) EtOH Reflux 6 h	n.s.	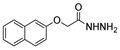 **8ak** (46%)
[43]	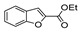 **17al**	Hydrazine 80% (n.s.) EtOH Reflux 6 h	n.s.	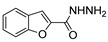 **8al** (57%)
[43]	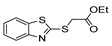 **17am**	Hydrazine 80% (n.s.) EtOH Reflux 6 h	n.s.	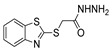 **8am** (56%)
[72]	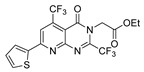 **17ao**	Hydrazine hydrate (~3 eq) EtOH Reflux 12 h	-	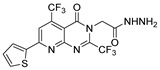 **8ao** (n.s.)
[73]	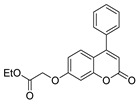 **17ap**	Hydrazine hydrate (2 eq) EtOH Reflux 8 h	-	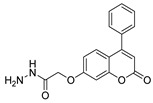 **8ap** (80%)
[75]	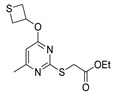 **17aq**	Hydrazine hydrate 85% (3 eq) EtOH r.t. 4 h	Recrystallization from isopropyl alcohol	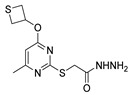 **8aq** (67%)
[76]	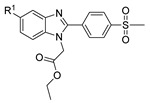 **17ar**	Hydrazine hydrate 99% (1 eq) EtOH Reflux 6 h	Recrystallization from ethanol	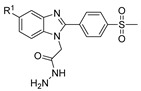 **8ar** R^1^ = H, Cl, CH_3_ (67–73%)
[77]	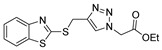 **17as**	Hydrazine hydrate (1.5 eq) EtOH Reflux 4 h	Recrystallization from ethanol	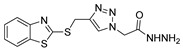 **8as** (90%)
[78]	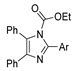 **17at**	Hydrazine hydrate (1.02 eq) EtOH Reflux 3 h	Recrystallization from ethanol or methanol	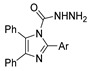 **8at** (89–97%)
[79]	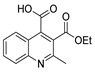 **17au**	Hydrazine hydrate (20 eq) EtOH Reflux 4 h	Recrystallization from ethanol	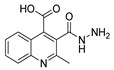 **8au** (61%)
[37]	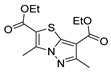 **17a**v	Hydrazine hydrate 80% (3 eq) EtOH Reflux 3 h	Recrystallization from ethanol/DMF	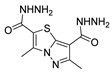 **8v** (90%)
[80]	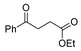 **17ac**	Hydrazine monohydrate (1 eq) EtOH Reflux 4 h	Recrystallization from dioxane	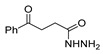 **8ac**(90%)
[81]	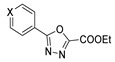 **17w**	Hydrazine hydrate (4 eq) EtOH Reflux 6 h	-	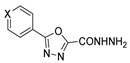 **8aw** X = N, CH (48–55%)
[82]	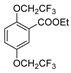 **17x**	Hydrazine (1 eq) EtOH Reflux 3 h	-	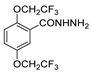 **8ax**(94%)
[83]	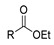 **17ay** R = 4-F-C_6_H_4_, **17az** R = 4-CH_3_–C_6_H_4_ **17aaa** R = 2-Cl,4-Cl-C_6_H_3_	Hydrazine hydrate (n.s.) EtOH Reflux n.s.	n.s.	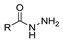 **8ay** R = 4-F-C_6_H_4_, **8az** R = 4-CH_3_–C_6_H_4_ **8aaaR** = 2-Cl,4-Cl-C_6_H_3_ (n.s.)
[84]	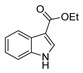 **17aab**	Hydrazine hydrate (10 eq) EtOH Reflux 7 h	-	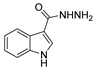 **8aab** (80%)
[2]	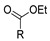 **17aac** R = 2-furyl **17aad** R = 3,4,5-(MeO)_3_C_6_H_2_ **17aai** R = 3,4-(MeO)_2_C_6_H_3_	Hydrazine hydrate (n.s.) - Reflux n.s.	-	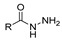 **8aac** R = 2-furyl **8aad** R = 3,4,5-(MeO)_3_C_6_H_2_ **8aai** R = 3,4-(MeO)_2_C_6_H_3_ (n.s.)
[85]	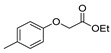 **17al**	Hydrazine hydrate (~11 eq) EtOH r.t. 3–4 h	-	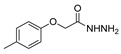 **8al** (98%)
[86]	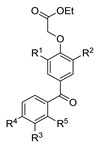 **17aaj**	Hydrazine hydrate (1.2 eq) EtOH r.t. 5–6 h	Recrystallization from ethanol	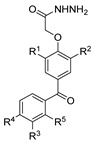 **8aaj** R^1^ = H, CH_3_, F; R^2^ = H, Cl; R^3^ = H, Cl; R^4^ = H, Cl, I; R^5^ = H, Cl (92% as an example)
[87]	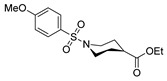 **17aak**	Hydrazine (1 eq) MeOH Reflux 2 h	-	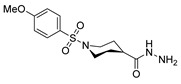 **8aak** (n.s.)
[42]	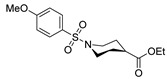 **17aal**	Hydrazine hydrate (n.s.) MeOH Reflux 4 h	-	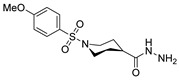 **8aal** (n.s.)
[88]	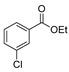 **17aam**	Hydrazine hydrate 80% (1 eq) Neat r.t. 4–5 h	-	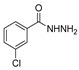 **8p** (96%)
[89]	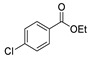 **17aan**	Hydrazine hydrate 80% (~11 eq) Neat r.t. 5–6 h	-	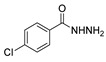 **8q** (96%)
[90]	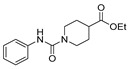 **17aao**	Hydrazine monohydrate (~34 eq) Neat r.t. 4–5 h	-	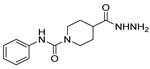 **8aao** (97%)
[91]	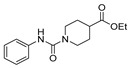 **17aao**	Hydrazine monohydrate 80% (~20 eq) Neat r.t. 4–5 h	-	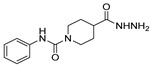 **8aao** (98%)
[92]	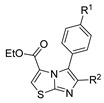 **17aap**	Hydrazine hydrate (10 eq, dropwise) EtOH and chloroform r.t. 24 h	Recrystallization from ethanol/water (60:40)	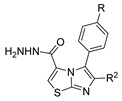 **8aap** R^1^ = Me, OMe, Br; R^2^ = Alk (72–80%)
[44]	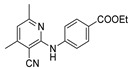 **17aaq**	Hydrazine hydrate (2 eq) EtOH Reflux 8 h	Recrystallization from ethanol	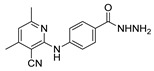 **8aaq** (52%)
[93]	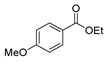 **17aar**	Hydrazine hydrate (~4 eq) MeOH Reflux 6 h	Recrystallization from methanol	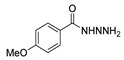 **8aar** (92%)
[94]	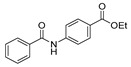 **17aas**	Hydrazine hydrate (n.s.) EtOH Reflux n.s.	n.s.	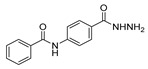 **8aas** (n.s.)
[95]	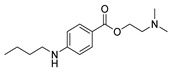 **17aat**	Hydrazine hydrate 80% (32.6 eq) EtOH Reflux n.s.	Recrystallization from ethanol	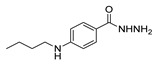 **8aat** (n.s.)
[96]	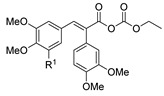 **18**	Hydrazine hydrate (n.s.) THF r.t. 4 h	n.s.	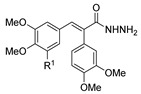 **8aau** R^1^ = Alk (80–95%)
[2]	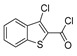 19a	Hydrazine hydrate (n.s.) - Reflux n.s.	-	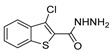 **8aav** (n.s.)
[97]	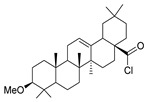 **19b**	Hydrazine hydrate (2 eq) DCM 25 °C Overnight	Silica gel column chromatography	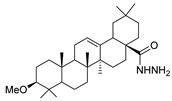 **8aaw** (89%)
[98]	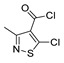 **19c**	1. Acetone NaN_3_ (aq.) 8 °C, 30 min 2. Anhydrous hydrazine (4 eq) 2-propanol Reflux 45 min	-	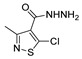 **8aax** (57%)
[99]	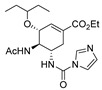 **20**	Hydrazine hydrate (2.2 eq) Et_3_N (1.5 eq), Na_2_SO_4_, CHCl_3_ 1. r.t 2. 35 °C. 1. 30 min 2. Overnight	-	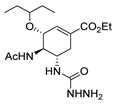 **8aay** (92%)
[28]	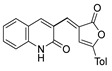 **21**	Hydrazine hydrate (1 eq) [100] EtOH r.t. 1 h	Recrystallization from ethanol	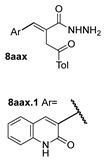 **8aax.1** (81%)
[101]	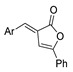 **22**	Hydrazine hydrate (1.1 eq.) EtOH r.t. n.s.	-	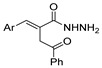 **8aba** (n.s.)
[103]	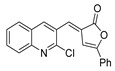 **23**	Hydrazine hydrate 85% (1 eq added dropwise) EtOH r.t. n.s.	Recrystallization from ethanol	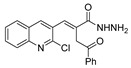 **8abb.1** (78%)

n.s.—not specified by the authors.

**Scheme 4:**
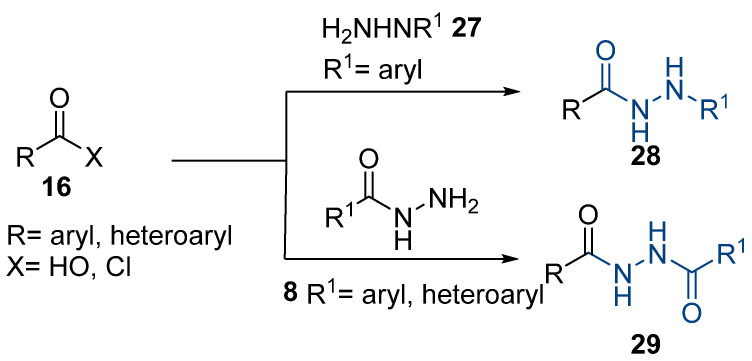
Representative scheme of substituted hydrazide synthesis.

Aside from the synthesis of unsubstituted hydrazides, substituted ones can be synthesized as illustrated in Scheme 4 and outlined in Table 2. In these cases, acids [106,107,108,109] or acyl halides [92,110] **16** react with hydrazides **8** or hydrazines **27** to acylate the most nucleophilic amine unit. When an acid is used as the starting material, the reaction is performed in the presence of a coupling agent, such as 1-ethyl-3-(3-dimethylaminopropyl)carbodiimide (EDC) [106,107,109]. Generally, products **28** and **29** were obtained in low to good yields (35–97%).

**Scheme 5:**
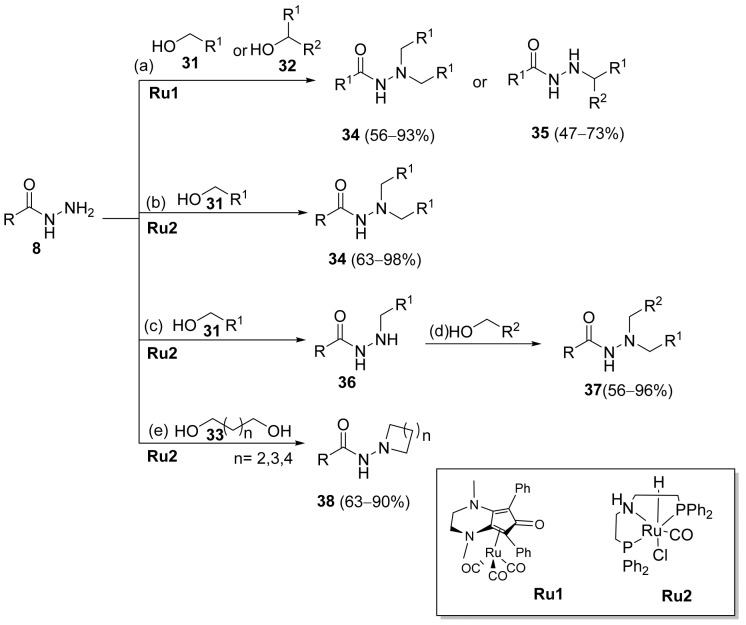
Synthesis of dialkylated and monoalkylated hydrazides; (a) alcohol (5 eq), Ru1 catalyst (1 mol%), Me_3_NO (2 mol%), NaO^t^Bu (0.5 eq), t–BuOH (0.5 M), 130 °C, 24 h; (b) alcohol (2.2 eq), Ru2 catalyst (2 mol%), KO^t^Bu (5 mol%), toluene, 135 °C, 24 h; (c) alcohol (1.1 eq), Ru2 catalyst (1 mol%), KO^t^Bu (5 mol%), toluene, 135 °C,12 h; (d) alcohol (1.1 eq), Ru2 (1 mol%), KO^t^Bu (5 mol%), toluene, 135 °C,12 h; (e) diol (2 eq), Ru2 catalyst, KO^t^Bu (5 mol%), toluene, 135 °C, 24 h.

Substituted hydrazides were also obtained via the alkylation of hydrazides **8** by reaction with primary or secondary alcohols as alkylating agents, under ruthenium complex catalysis (Scheme 5). Joly et al. [111] reported the synthesis of dialkylated and monoalkylated hydrazides **34** or **35**, respectively, in reasonable to excellent yields using diaminocyclopentadienone ruthenium tricarbonyl complex **Ru1**. On the other hand, Thiyagarajan et al. [112] reported the synthesis of both symmetrical and unsymmetrical *N*,*N*-disubstituted hydrazides **34** and **37.** The use of diols led to the intramolecular cyclization of acylhydrazides to generate **38**.

**Scheme 6:**
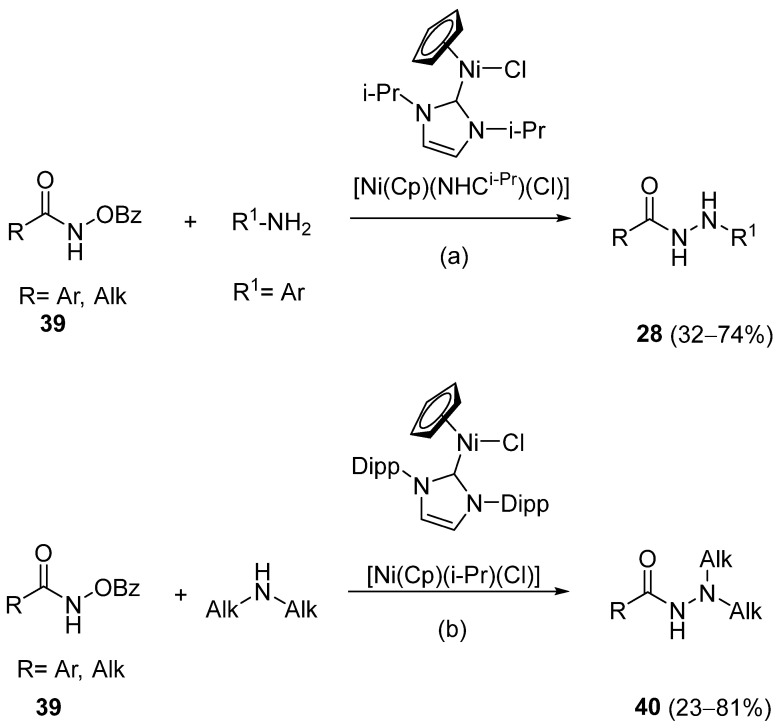
Synthesis of substituted hydrazides from hydroxamates. (a) Ni catalyst (10 mol%), PhSiH_3_ (1 eq), 1:4 CH_2_Cl_2_/THF (0.2 M), 30 °C, 24 h; (b) Ni catalyst (10 mol%), PhSiH_3_ (1 eq), MSTFA (1.5 eq), 1:4 CH_2_Cl_2_/THF (0.2 M), 30 °C, 24 h.

Barbor et al. [113] reported a novel nickel-catalyzed method for the synthesis of hydrazides **28** and **40** through N-N cross-coupling reactions from hydroxamates **39** in the presence of aromatic or aliphatic amines. These reactions occurred by catalysis with the nickel complex presented in Scheme 6. According to the authors, N-N coupling occurs efficiently when hydroxamates with electron-donating or -withdrawing *para*-substituents are used together with aniline derivatives. The aniline derivatives may have *para*-electron-donating or sterically hindered *ortho* substituents, such as halide (with no indication of protodehalogenation) or alkyl groups. Additionally, the reaction is also compatible with aniline derivatives bearing unprotected ketone and hydroxyl moieties. Primary, secondary, and tertiary aliphatic hydroxamates were also well tolerated. Moreover, in the presence of secondary aliphatic amines, in situ silylation allows N-N coupling in low to good yields [113].

**Scheme 7:**
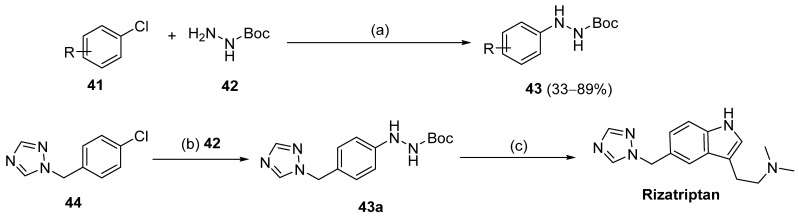
Synthesis of **43** and rizatriptan: (a) (NiBr_2_·glyme, d-Mebpy) (10 mol%) purple LEDs (390–395 nm), Cy_2_NMe, TBAI, CH_3_CN, 80 °C, Ar, 12 h; (b) **42** (NiBr_2_·glyme, d-Mebpy), purple LEDs (390–395 nm) Cy_2_NMe, TBAI, CH_3_CN, 80 °C, Ar, 24 h; (c) i. 4 M HCl, THF, r.t., 6 h; ii. Me_2_N(CH_2_)_3_CH(OEt)_2_, HCl, H_2_O, 70 °C, 2 h.

On the other hand, nickel-catalyzed photochemical C-N coupling reactions were performed by Li et al. [114] to react (hetero)aryl halides **41** with hydrazides/N-Boc hydrazine **42** (Scheme 7). This arylation reaction was catalyzed by a Ni(II)–bipyridine complex in the presence of purple LEDs. The reaction of N-Boc hydrazine with aryl chlorides **41** with electron-deficient and electron-neutral substituents at the para position yielded aryl hydrazides **43** with low to good yields (33–89%). Also, the synthesis of rizatriptan was conducted after applying this method for the synthesis of intermediate **43a** [114].

**Scheme 8:**
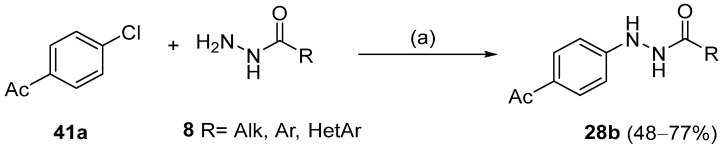
Synthesis of hydrazides **28b**: (a) (NiBr_2_·glyme, d-Mebpy) (10 mol%) purple LEDs (390–395 nm), Cy_2_NMe, TBAI, CH_3_CN, 80 °C, Ar, 12 h.

Moreover, many alkyl and aryl hydrazides **8** were coupled with 4-chloroacetophenone **41a**, yielding hydrazides **28b** generally in good yields (Scheme 8). Aryl hydrazides with electron-rich and electron-neutral substituents and heteroaryl hydrazides were well tolerated in this reaction (48–77%) [114].

**Scheme 9:**
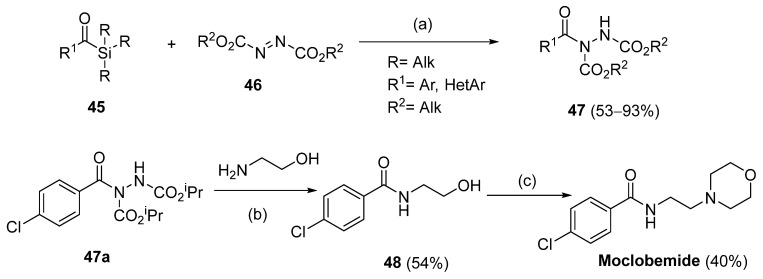
Synthesis of hydrazides **47** and moclobemide: (a) toluene, 456 nm, rt, 16 h; (b) DCM, r.t., 20 h; (c) i. MsCl, DCM, Et_3_N, DMAP, r.t., 24 h; ii. morpholine, 100 °C, 6 h.

Hydrazides **47** were obtained from substituted azodicarboxylates **46** and arylic or heteroarylic acylsilanes **45** in the presence of visible light. This reaction afforded hydrazides **47** in good yields (53–93%). The drug moclobemide was obtained from the precursor **47a**, which was synthesized by the previous method (Scheme 9) [115].

**Scheme 10:**
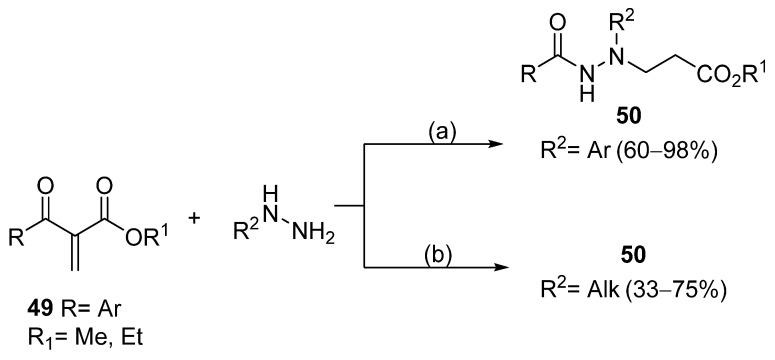
Synthesis of compounds **50**; (a) Et_3_N (0.5–3.5 eq), CH_3_CN, r.t., 30 min–15 h; (b) Et_3_N (3.5 eq), H_2_O, CH_3_CN, r.t., 24 h.

*N,N*′-disubstituted benzohydrazides **50** were obtained from benzoyl acrylates **49** via a hydrazine insertion into the β-position through an unexpected carbon–carbon bond cleavage (Scheme 10) [116].

### 2.2. Biological Activity of Hydrazides

**Figure 3:**
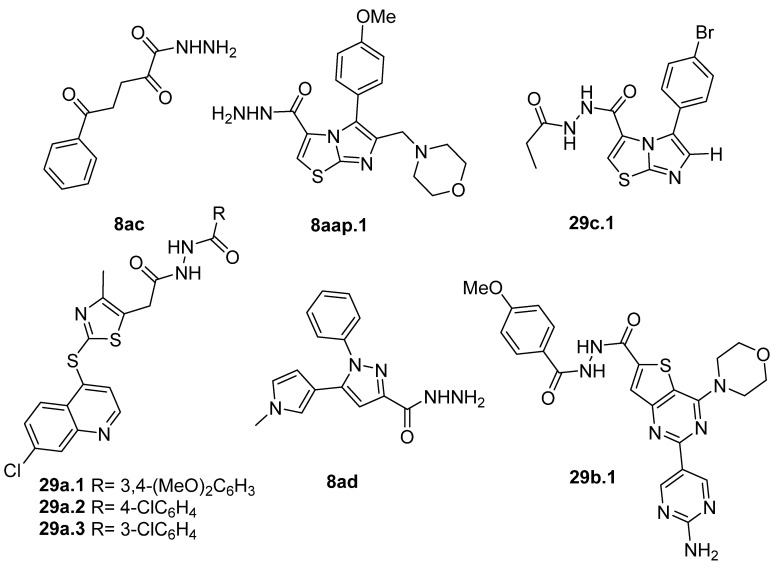
Hydrazides with anticancer activity.

The compounds presented in Figure 3 showed anticancer activity against various cancer cell lines. Hydrazide **8ac** showed good anticancer activity against MCF-7 breast cancer and HepG2 hepatocellular carcinoma cell lines with IC_50_ = 8.1 µM and IC_50_ = 28.6 µM, respectively [64]. Hydrazides **8aap.1** and **29c.1** also displayed anticancer activity towards the MCF-7 cancer cell line with IC_50_ values of 2.37 and 1.83 µM, respectively [92]. Besides that, Sabry et al. [92] reported that these hydrazides showed a strong dual inhibition activity of EGFR/HER2 kinase with IC_50_ values of 0.153 µM (EGFR) and 0.108 µM (HER2) for **29c.1** and 0.122 µM (EGFR) and 0.108 µM (HER2) for **8aap.1**. In in vivo studies in Swiss albino mice mammary glands, compounds **8aap.1** and **29c.1** showed tumor volume reductions by 65.3 and 76.5%, respectively, at 10 mg/kg.

Derivatives **29a.1** and **29a.2**, reported by Ramírez et al. [106], were also tested against the MCF-7 cancer cell line. Compounds **29a.1** and **29a.2** presented IC_50_ values of 15.41 and 12.99 µM, respectively. In addition, derivatives **29a.3** and **29a.2** were active against the A549 cell line (lung cancer) with IC_50_ values of 37.17 and 31.02 µM, respectively [106].

Hydrazides **8ad** and **29b.1**, reported by Abdelrehim et al. [35] and Han et al. [107], presented activity against the HCT-116 colorectal cancer cell line with IC_50_ values of 8.44 µg/mL and 2.02 µM (Figure 3). Compound **29b.1** also showed activity against PC-3 (prostatic adenocarcinoma), A549 (lung cancer), and MDA-MB-231 (triple-negative breast cancer) cancer cell lines, with IC_50_ values of 1.95, 1.62, and 1.55 µM. It also showed potent inhibitory activities against phosphatidylinositol 4,5-bisphosphate 3-kinase catalytic subunit alpha isoform (PI3Kα) with an IC_50_ = 0.46 nM and mammalian targeting of rapamycin (mTOR) with an IC_50_ = 12 nM [107].

**Figure 4:**
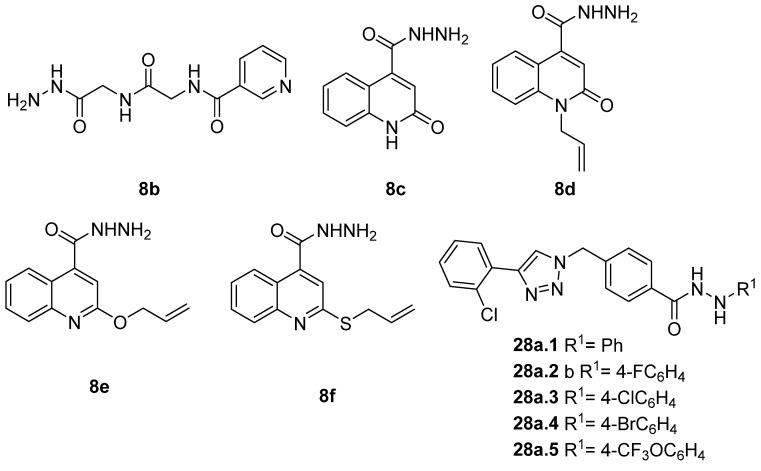
Hydrazides with antibacterial and antifungal activity.

Hydrazide **8b** (Figure 4) was evaluated for its antibacterial and antifungal activities. It showed a strong antibacterial and antifungal activity, with inhibition zones of 29, 30, 28 and 16 mm against *Bacillus subtilis*, *Escherichia coli*, *Candida albicans* and *Aspergillus niger*, respectively [51]. Compounds **8c**–**8f** also exhibited activity against *E. coli*, *B. subtils*, and *Asp. niger* strains, presenting inhibition zones varying between 2 and 5 mm [26].

Compounds **28** (Figure 4) were evaluated as fungicides [109]. Compounds **28a.1**–**28a.5** exhibited growth inhibition activity against *Botryosphaeria dothidea*, *Rhizoctonia solani*, and *Gibberella zeae* with EC_50_ values within the 10.0–0.306 µg/mL range, which were higher activity than those of the commercial agrochemicals azoxystrobin, boscalid, and fluxapyroxad [109].

**Figure 5:**
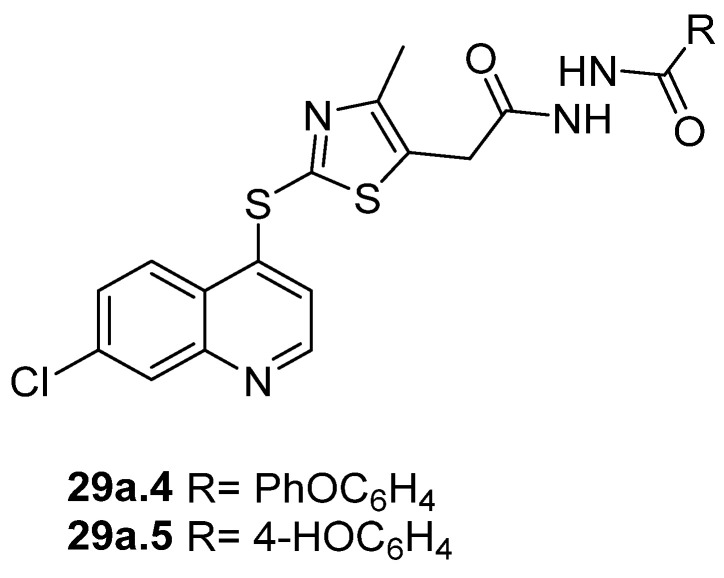
Hydrazides with antiparasitic activity.

Disubstituted hydrazides **29** were tested as antimalarial agents. Compounds **29a.4** and **29a.5** (Figure 5) showed antimalarial activity with IC_50_ values of 0.65 and 0.64 µM, respectively [106].

**Figure 6:**
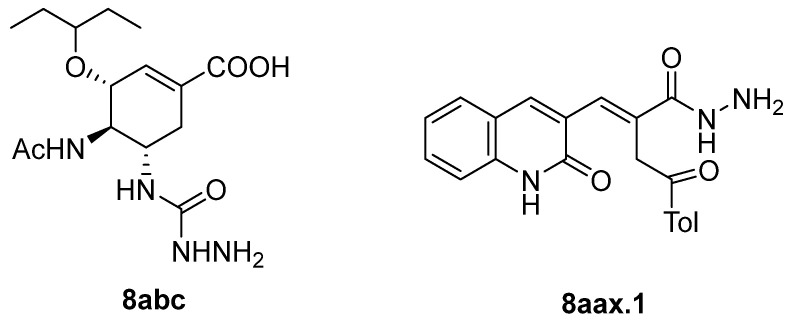
Hydrazides with antiviral activity.

Hydrazides present in Figure 6 were evaluated as antivirals. Compound **8abc** showed antiviral activity against influenza A as a Neuraminidase inhibitor against H5N1 and H1N1 subtypes with IC_50_ values of 26.8 nM and 11.9 nM, respectively [99]. Moreover, hydrazide **8aax.1** was presented as a great immunomodulator, presenting 80% protection against the highly pathogenic avian influenza virus (H5N8) [28].

**Figure 7:**
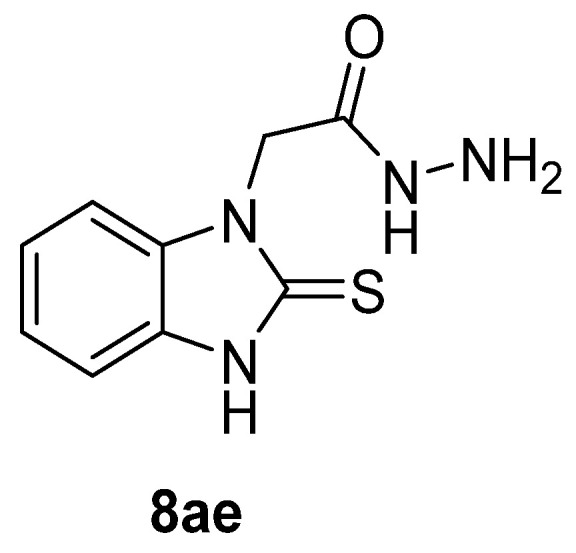
Myeloperoxidase inhibitor.

Myeloperoxidase plays a key role in the human antimicrobial system by oxidizing vital molecules of microorganisms in phagolysosomes through the production of hypochlorous acid. It has been associated with inflammatory diseases such as renal injury, multiple sclerosis, and cardiovascular and neurodegenerative diseases. Saylam et al. [65] reported compound **8ae** (Figure 7) as an excellent myeloperoxidase inhibitor with an IC_50_ = 0.393 µM, which is comparable to the standard drug 4-aminobenzoic acid hydrazide.

## 3. Hydrazide Derivatives

### 3.1. Hydrazide–Hydrazones

#### 3.1.1. Synthesis of Hydrazide–Hydrazones

Hydrazide–hydrazone derivatives are among the most frequently synthesized and reported hydrazide derivatives in the literature. These compounds stand out in organic and medicinal chemistry since they have exhibited a wide range of biological activities and have been used as important intermediates in the synthesis of heterocycle rings from hydrazides. The hydrazide–hydrazone moiety contains the functional group -CO-NH-N=CR^1^R^2^, which is a combination of the hydrazide and imine groups. The imine group confers E/Z isomerism and photochromism in both solution and the solid state [55,97]. Moreover, the -NH- and C=O groups allow the compounds to have the capability of binding to anions/cations and biomolecules; the coexistence of imine and carbonyl groups allows them to establish metallo-assemblies [55].

In the past few years, hydrazides have been extensively used to synthesize several hydrazide–hydrazone derivatives, as potential aggregation-induced emission luminogens (AIEgens), probes, or anticancer, antimicrobial, antifungal, antituberculosis, antimalarial, antiviral, and antioxidant agents. Some derivatives exert their activity through the inhibition of specific enzymes such as acetylcholinesterase, butyrylcholinesterase, α-glucosidase, and others.

**Scheme 11:**
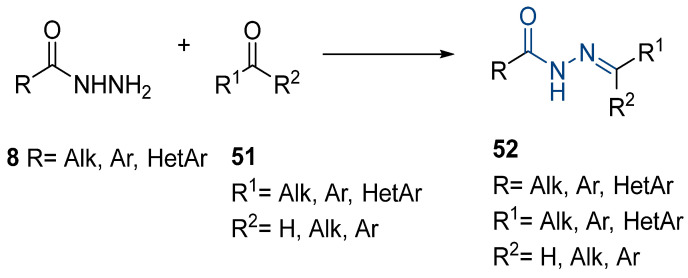
Representative scheme of hydrazide–hydrazones synthesis.

Hydrazide–hydrazones **52** are synthesized from the reaction between a hydrazide and an aldehyde/ketone [13,34,37,38,43,53,54,56,57,66,67,68,71,72,73,75,76,77,78,79,95,96,97,98,101,117,118,119,120,121,122,123,124,125,126,127] (Scheme 11). According to the studies in this review, these reactions, in general, occur in alcohols (ethanol or methanol) and at high temperatures (Table 3). The reactions occurred without or with acid catalysis, such as acetic acid [34,43,54,66,71,73,76,77,79,95,96,117,119,121,123,127] or *p*-TsOH [55,56], and in these cases, the reactions may occur at room [55,56,79,117] or high temperatures [34,38,43,54,66,68,71,72,73,76,77,95,96,119,121,123,127]. Hydrazide–hydrazone derivatives were obtained in low to excellent yields.

As mentioned earlier, some hydrazides are commercially available. However, others are synthesized by the scientific community to originate the required compounds. Here, hydrazide–hydrazones synthesized from alkyl, aryl, or heteroaryl hydrazides as starting materials, produced or not by the authors, will be presented. The biological activity of the generated compounds will also be reviewed.

#### 3.1.2. Biological Activity of Hydrazide–Hydrazones

**Figure 8:**
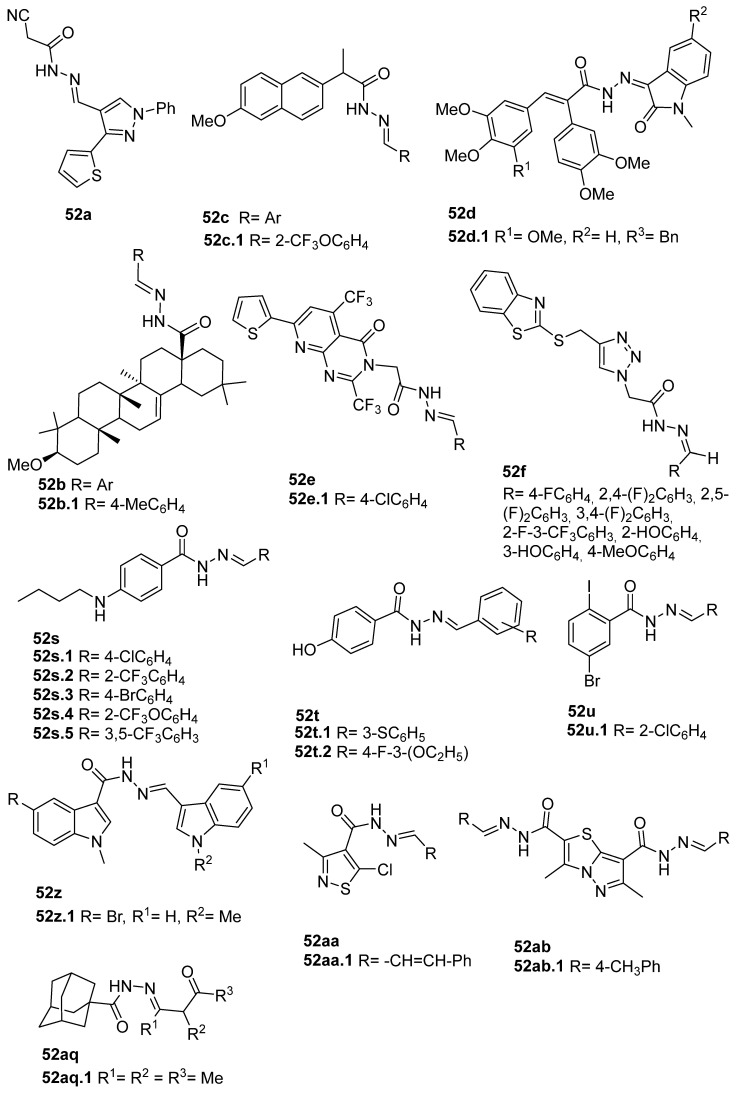
Hydrazide–hydrazones with anticancer activity.

The new hydrazide–hydrazone derivatives represented in Figure 8 were evaluated as anticancer agents. Compounds **52a**, **52c.1**, **52d.1**, and **52e.1** showed good activity against human breast cancer cell lines, specifically against the MCF7 line, with IC_50_ values of 7.38, 59.81, 3.49, and 14.6 µM, respectively [68,72,96,124]. Moreover, compound **52c.1** also showed promising anticancer activity, with IC_50_ = 22.42 µM, against the human breast cancer cell line MDA-MB-231. Compound **52c.1** was tested in vivo and decreased the tumor volume in both low (60 mg/kg) and high (120 mg/kg) doses in mice [68]. Besides the activity against breast cancer, compound **52a** showed activity against the HepG2 cancer cell line with IC_50_ = 8.79 µM [124]. In addition, derivatives **52d** were also tested against HCT-116 and SK-MEL-28 (melanoma) cancer cell lines. Compound **52d.1** displayed the highest activity with IC_50_ values of 6.82 and 10.39 µM, respectively, with no relevant toxicity on non-malignant HaCaT (human keratinocyte) cells [96].

According to Halil et al. [97], natural compounds with bioactive properties, when combined with hydrazides, can lead to new active compounds with increased activity. Hence, Halil et al. [97] synthesized molecules with structure **52b** (Figure 8) starting from the natural product oleanolic acid. The in vitro anticancer activity was studied on the A549 (adenocarcinomic human alveolar basal epithelial) cell line. Of the thirteen compounds synthesized, compound **52b.1** showed the best activity with IC_50_ = 0.08 µM and low cytotoxicity on the BEAS-2B cells (human non-tumorigenic lung epithelial cells).

The anticancer activity of combretastatin–oxindole **52d**, pyrimidine derivatives **52e**, and triazoles **52f** (Figure 8) was also evaluated against the A549 cell line. Compounds **52d.1** and **52e.1** were promising anticancer agents with IC_50_ values of 1.26 and 11.3 µM, respectively [72,77,96]. Furthermore, Abba et al. [72] identified the derivative **52e.1** as a potent compound against DU145 (prostate cancer) using HeLa (cervical cancer) cell lines with IC_50_ values of 13.4 and 9.1 µM, respectively [72].

According to Almehmadi et al. [77], molecules **52f** revealed an anticancer capacity, presenting a growth inhibition ranging from 55 to 90% at 400 µg/mL against the A549 cell line.

Han et al. [95] described derivatives 52s.1 and 52s.2 with high anticancer activity against the human colorectal adenocarcinoma (Colo-205) cell line (IC_50_ = 50.0 and 20.5 µM, respectively). On the other hand, compounds 52s.3, 52s.4, 52s.5, 52t.1, and 52t.2 displayed the great anticancer activity against the liver hepatocellular carcinoma HepG2 cell line with IC_50_ = 30.5, 35.9, 20.8, 42.4, and 37.4 µM, respectively [66]. Derivatives 52t.1 and 52t.2, reported by Han et al. [66] (Figure 8) exhibited lower activity than derivatives 52s. Among the thirteen different hydrazones 52u, described by Popiołek et al. [67], compound 52u.1 exhibited the best cytotoxicity with IC_50_ = 33.45 and 11.94 µM against hepatocellular carcinoma (HepG2) and renal adenocarcinoma (769-P) cell lines, respectively, and additionally showed high selectivity, with low cytotoxicity against the normal Vero cell line, with IC_50_ = 320.54 µM.

Among indole derivatives **52z** [34], compound **52z.1** was the most active against the A549 lung adenocarcinoma cell line with IC_50_ = 0.793 µM. This compound also showed great activity against cervical HeLa and breast MCF-7 cancer cells with IC_50_ = 1.69 and 1.19 µM. The authors studied the mechanisms of action of compound **52z.1** regarding different signaling pathways triggered in HeLa and MCF-7 cells, and it was verified that this compound induced cell apoptosis through the generation of reactive oxygen species and activation of many signal transduction pathways [34].

Thiazole derivatives **52aa** (Figure 8) were screened towards various cancer cell lines, and **52aa.1** exhibited the highest antiproliferative activities with IC_50_ = 14, 25, 34.2, 39.3, and 68.6 µM against human leukemia MV4-11 cells, colon LoVo and LoVo/DX, and breast MCF-7 and MCF-10A cancer cell lines, respectively [98].

Alsayari et al. [37] reported compounds **52ab** (Figure 8), from which **52ab.1** presented the highest activity towards HepG-2 and HCT-116 cell lines with IC_50_ = 30.5 and 86.9 µg/mL, respectively.

Adamantane-1-carbohydrazone derivatives **52aq** were tested as anticancer agents for breast, liver, and lung cancers. Derivative **52aq.1** stood out with IC_50_ = 8.35, 7.82 and 4.39 µM, for MCF-7, HepG-2 and A549 cell lines, respectively [127].

**Figure 9:**
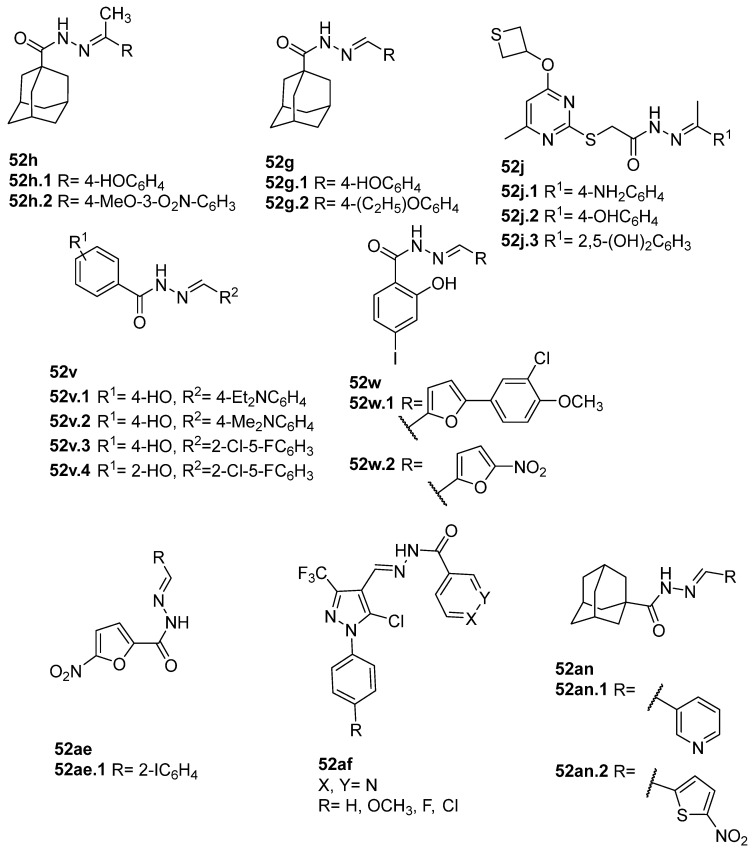
Hydrazide–hydrazones with antibacterial, antifungal, or antiparasitary activity.

Compounds **52h** and **52g** (Figure 9) were evaluated for their antimicrobial activity against *Enterococcus faecalis*, *Staphylococcus aureus, Bacillus cereus*, and *Candida albicans*. Compounds **52h.1** presented an MIC of 12.5 µM for the four strains, and **52h.2** presented IC_50_ values of 6.35 µM, 6.77 µM, 6.12 µM, and 6.37 µM against the same strains. Compounds **52g.1** and **52g.2** showed good inhibitory activity with IC_50_ values of 3.56 and 6.73 µM against *Enterococcus faecalis* and 6.77 and 6.66 µM against *Candida albicans*, respectively [57].

Pyrimidine derivatives **52j.1**–**52j.3** (Figure 9) also exhibited high antimicrobial activities with MIC = 0.05 µg/mL against *St. aureus*, *Str. Pyogenes*, *P. vulgaris*, *K. pneumoniae*, *Ent. Aerogenes*, *P.S aeruginosa*, and *C. Albican* [75].

Compounds **52v** and **52w** (Figure 9) showed antibacterial potential against *S. aureus*, *E. Coli*, and *B. subtilis*. Specifically, compound **52v.1** was the best antibacterial agent against *S. aureus* with an MIC value of 0.625 µg/mL. Compounds **52v.1**–**52v.4** had higher activity against *E. coli* with MIC = 0.625 µg/mL. Moreover, compounds **52v.3** and **52v.4** displayed the best activities against *B. subtilis* with MIC = 0.312 µg/mL [118]. Popiołek et al. [38] reported compounds **52w.1** and **52w.2** with MIC = 7.81 µg/mL towards *B. subtilis* (ATCC 6633).

In 2020, furyl hydrazide–hydrazones **52ae** were also tested for their potential antimicrobial activity [53]. The assays of antibacterial and antifungal activity revealed that several of the synthesized compounds **52ae** (Figure 9) presented very strong bioactivity with MIC < 10 µg/mL. Some compounds showed higher activity than the standard drugs (nitrofurantoin, cefuroxime, and ampicillin). For example, compound **52ae.1** was 130 times more active towards *Bacillus subtilis* ATCC 6633 (MIC = 0.48 µg/mL) than ampicillin [53].

The novel derivatives **52af** (Figure 9) were evaluated for their inhibitory effects in several bacterial and fungal strains [120]. These compounds exhibited the maximum zone of inhibition ranging from 7 to 20 mm against *B. licheniformis and S. aureus*, as well as good antifungal activity against both *Asp. niger* and *C. albicans* with maximum zones of inhibition of 8–14 and 6–14 mm, respectively [120].

Adamantane hydrazide–hydrazone derivatives **52an.1** and **52an.2** presented by Al-Wahaibi et al. [125] also showed good antibacterial activity. Compound **52an.2** showed broad-spectrum antibacterial and antifungal activity with MIC = 1.5 µM against *St. aureus* and *B. subtilis,* MIC = 3 µM against *M. luteus*, and MIC = 6 µM against *E. coli* and *P.S aeruginosa*. Compound **52an.1** only showed potent activity against the tested Gram-positive bacterial strains, with MIC = 3.53 uM against *St. aureus* and *B. subtilis* and MIC = 7.06 uM for *M. luteus* [125].

**Figure 10:**
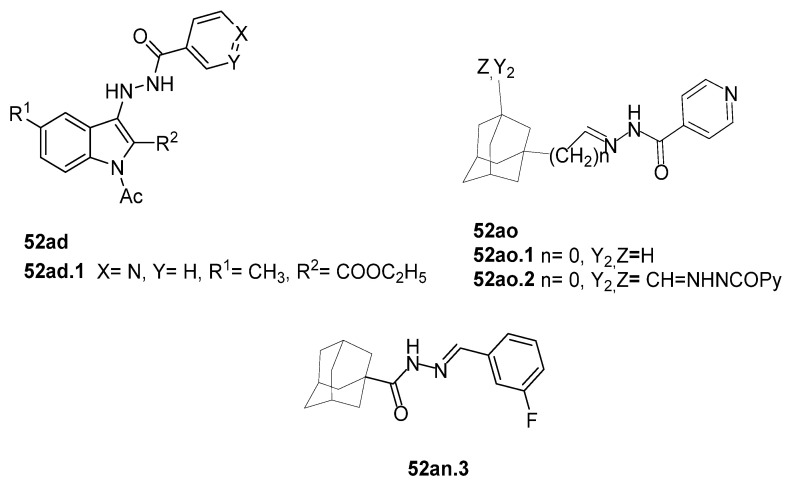
Hydrazide–hydrazones with antimycobacterial activity.

In the literature, simpler heteroaryl hydrazides, like isonicotinic hydrazide, or similar ones, have been employed to obtain compounds with a wide range of biological activities (Figure 10) [119,121,123,128]. A series of derivatives **52ad** that combine pyridine and indole moieties were reported, and the in vitro antimycobacterial activity was studied against strain *M. tuberculosis* H37Rv and against a clinical isolate of isoniazid-resistant *M. tuberculosis* strain, designated as CN-40 [119]. The hydrazide hybrid **52ad.1** (Figure 10) was the most promising compound with MIC = 0.05 µg/mL and with a high selectivity index (SI = 300). Compared to isoniazid **1**, the new compound **52ad.1** had similar activity against *M. tuberculosis* H37Rv; still, the new compound showed higher activity than isoniazid against the isoniazid-resistant *M. tuberculosis* CN-40 strain [119]. Papageorgiou et al. [126] reported new isoniazid-based adamantane derivatives, **52ao.1** and **52ao.2**, with activity against *M. tuberculosis* H37Rv, MIC = 0.14 and 0.09 nM, respectively, and very low cytotoxicity against HepG2 (selectivity index ≥ 2500).

On the other hand, adamantane hydrazide–hydrazone derivative **52an.3** was also reported as an antituberculosis agent exhibiting MIC values of 0.2, 0.3, 1.5, 12.5, and 12.5 µg/mL for *M. tuberculosis, M. bovis BCG*, *M. smegmatis*, *M. abscessus*, and *M. marinum*, respectively [129].

**Figure 11:**
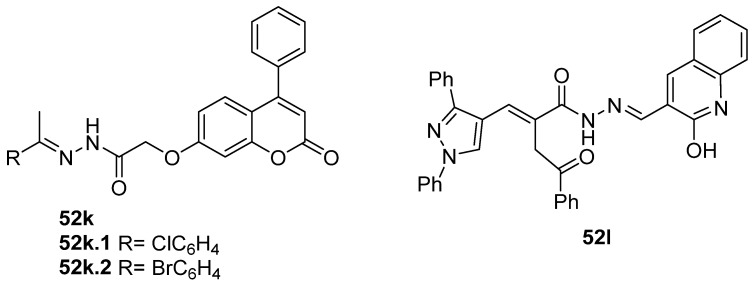
Hydrazide–hydrazones with antiviral activity.

Antiviral activity was detected for hydrazide derivatives represented in Figure 11. Kassem et al. [73] obtained the 4-phenylcoumarin derivatives **52k**, and these proved to be potential inhibitors of the 3C protease of the hepatitis A virus. Compounds **52k.2** and **52k.1** presented the highest effects against viral adsorption and replication with IC_50_ = 8.5 and 10.7 µg/mL, respectively. Morsy et al. [101] reported derivative **52l** that showed 100% protection against Newcastle disease virus.

**Figure 12:**
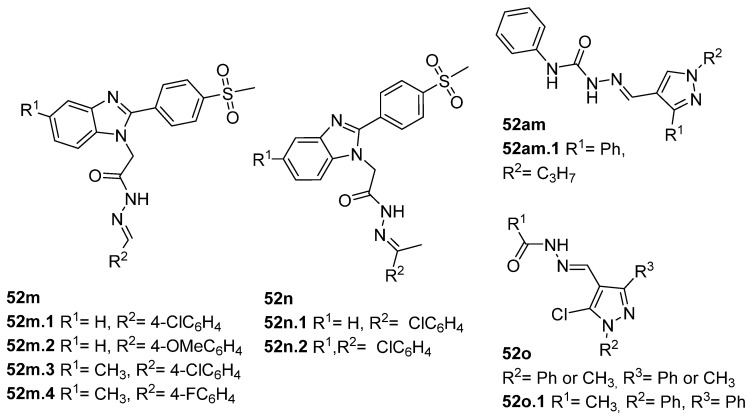
Hydrazide–hydrazones with anti-inflammatory activity.

A series of benzimidazole derivatives were synthesized and evaluated as cyclooxygenase-1(COX-1)/cyclooxygenase-2 (COX-2) inhibitors [76]. The results of the cyclooxygenase (COX) inhibition assay generally showed that the compounds **52m** and **52n.1** (Figure 12) exhibited low selectivity towards the COX-1 isozyme compared to those of reference drugs (indomethacin and celecoxib). Compounds **52m.1**, **52m.4**, **52n.1**, and **52n.2** showed selective inhibition of the COX-2 isozyme. Among all, compound **52m.1** exhibited the highest COX-2 inhibitory activity with an IC_50_ = 0.10 µM and selectivity index SI = 134 [76]. Compounds **52m.1**–**4**, **52n.1**, and **52n.2** also exhibited good anti-inflammatory activity, reducing inflammation by more than 93%. Regarding the ulcerogenic liability, compound **52m.1** was the safest one with an Ulcer Index (UI) = 0.83 and a lower ulcerogenic effect than the reference drugs celecoxib (UI = 3.5) and indomethacin (UI = 13) [76].

Tumor Necrosis Factor alpha (TNF-α) is a pro-inflammatory cytokine that may trigger and amplify inflammatory signals via multiple signaling pathways [130]. The dysregulation of inflammation, particularly the dysregulation of TNF-α, has been associated with various diseases such as arthritis, atherosclerosis, neurodegenerative diseases, diabetes, and cancer [131]. Therefore, inhibition of TNF-α leads to higher control and better treatment of inflammatory diseases [117]. In 2019, Liang et al. [132] synthesized pyrazole–hydrazone **52am.1**, which showed excellent TNF-α inhibitory activity, and some displayed comparable anti-inflammatory activity to dexamethasone (reference drug) in vivo. In continuation of this work, Song et al. [117] discovered new pyrazole–hydrazone derivatives **52o** (Figure 12), particularly compound **52o.1**, which inhibited TNF-α in a dose-dependent manner with IC_50_ = 5.56 µM. The authors also presented molecular docking results for **52o.1**, concluding that the compounds’ phenyl and hydrazide groups play an important role in binding to the target site [117].

**Figure 13:**
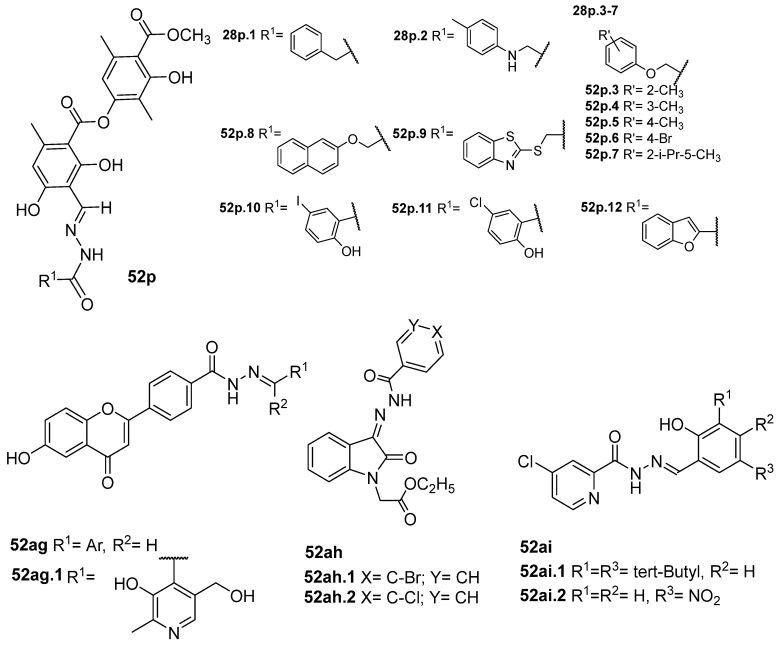
α-glucosidase, α-amylase, acetylcholinesterase, and butyrylcholinesterase inhibitors.

Duong et al. [43] obtained the compounds **52p** (Figure 13) by reacting atranorin (natural product) with several hydrazides. The compounds were tested for the inhibition of α-glucosidase. Compounds **52p1**–**12** exhibited stronger activities than the natural product and acarbose, with IC_50_ values ranging from 6.67 to 54.7 µM. Compound **52p.1** exhibited the best activity, with IC_50_ = 6.67 µM. Additionally, the cytotoxicity of these compounds against the normal cell line HEK293 was evaluated, and they exhibited weak to no cytotoxicity [43].

New flavone derivatives **52ag** and indolone derivatives **52ah** also exhibited inhibitory activity against α-glucosidase (Figure 13). Compounds **52ag.1** (IC_50_ = 1.02 µM), **52ah.1** (IC_50_ = 14.8 µg/mL), and **52ah.2** (IC_50_ = 14.5 µg/mL) showed higher activity than the standard drug acarbose [54,121].

Additionally, Abbasi et al. [121] reported compounds **52ah** as inhibitors of the α-amylase enzyme. Compounds **52ah.1** and **52ah.2** were the most potent against the α-amylase enzyme with IC_50_ = 19.6 and 18.3 µg/mL, respectively.

In addition, acetylcholinesterase (AChE) and butyrylcholinesterase (BChE) are other critical enzymes in health care, and their inhibitors are used in the clinical management of Alzheimer’s disease. AChE inhibitors include galantamine, donepezil, rivastigmine, and tacrine; however, these drugs have numerous side effects. So, Güngör [122] reported the synthesis of eleven new derivatives **52ai** (Figure 13) as potential inhibitors of AChE enzymes. Among these, compound **52ai.1** showed the best inhibitory activity with IC_50_ = 2.01 µM against AChE, comparable to the control Galantamine (IC_50_ = 2.60 µM). In addition, compound **52ai.2** showed the best inhibitory effect with IC_50_ = 2.83 µM against BChE, which was lower than the IC_50_ of control galantamine (IC_50_ = 3.70 µM) [122].

**Figure 14:**
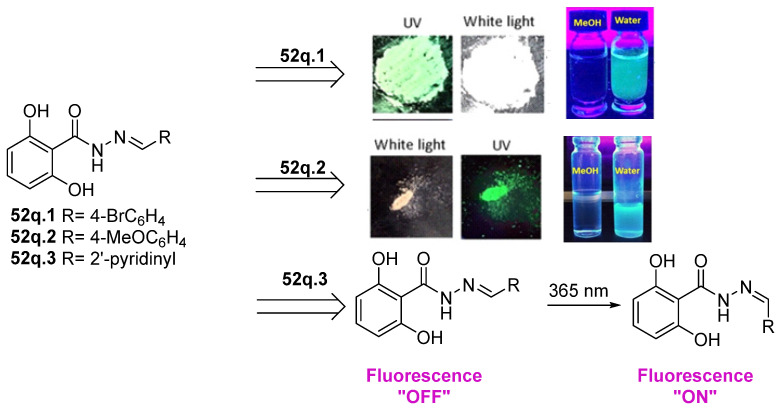
Hydrazide–hydrazones with aggregate-induced emission properties. Adapted with permission from Ref. [55] Copyright 2019 John Wiley & Sons, Inc., and Ref. [56] Copyright 2021 Royal Society of Chemistry.

Hydrazides with aryl-substituted groups have also been used to obtain hydrazide–hydrazones with aggregation-induced emission (AIE) properties. Patil et al. [55] reported novel hydrazide–hydrazone **52q** with remarkable AIE properties. The study showed that compounds **52q.1** and **52q.2** behaved as aggregate-induced emission luminogens to illuminate and record images of subcellular organelles and targets in cancer cells (Figure 14) [55]. Moreover, the internalization of these compounds into the HeLa cervical cancer cells without showing any cytotoxicity was observed. On the other hand, Wu et al. [56] reported that compound **52q.3** (the *E* isomer) exhibited photoisomerization to the Z isomer after light irradiation at 365 nm (Figure 14).

**Figure 15:**
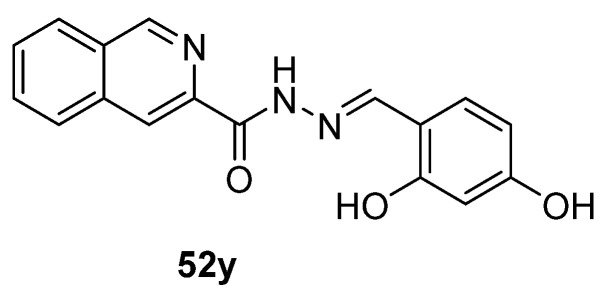
Hydrazide–hydrazone as potential probes.

Recently, the research of Fan et al. [13] highlighted a new fluorescent probe **52y** (Figure 15) capable of self-assembling into nanospheres in aqueous solution, and then, when placed in the presence of human serum albumin (HSA), they disassemble and display an evident fluorescence signal.

**Figure 16:**
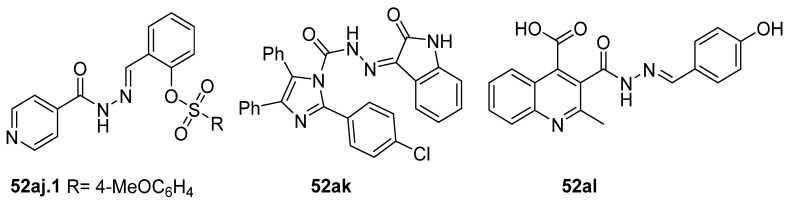
Hydrazide–hydrazone with antioxidant activity.

Compounds **52aj**, **52ak**, and **52al** (Figure 16) were tested as potential antioxidants [78,79,123]. Among the twelve derivatives **52aj** tested in vitro, compound **52aj.1** showed the best activity according to the DPPH method (SC_50_ = 0.03 mg/mL) [123]. However, compound **52ak** showed antioxidant activity in vivo in rats according to Abdelhamid et al. [78]. Amongst the quinoline hydrazide–hydrazone derivatives synthesized by Cahyana et al. [79], compound **52al** showed the best antioxidant activity by DPPH assay with IC_50_ = 843.52 ppm, yet this was weak compared to ascorbic acid with IC_50_ = 11 ppm.

Apart from their biological importance, hydrazide–hydrazone compounds are occasionally mentioned in the following points as useful intermediate synthons for the synthesis of some heterocyclic rings [40,47].

### 3.2. Heterocycles from Hydrazides

Hydrazides are widely used as synthons in the synthesis of a variety of heterocycles via electrophilic reactions. After the cyclization process, the different heterocycles can also be further modified or not to obtain compounds with biological activity. Within the heterocycles generated from hydrazides, it has become possible to identify the synthesis of pyrrolones, pyrazoles, oxadiazoles, thiadiazoles, triazoles, and triazepinones in the recent literature, which will be discussed in the following sections. The biological activity of the synthesized compounds will also be presented.

#### 3.2.1. Pyrrolones

##### Synthesis of Pyrrolones

Pyrrolones are five-membered heterocyclic lactams recognized as important scaffolds whose origin may be natural or synthetic, with a wide variety of pharmacological activities [133,134]. These compounds can present anticancer [27,135,136,137], antimalarial [138], anti-inflammatory [139], antiviral [28], and antioxidant activities [140]. In 2015, Pelkey et al. [141] reported different methods, including one-component intramolecular or two-component intermolecular cyclization approaches for pyrrolone synthesis that were reported through the end of 2014.

**Scheme 12:**
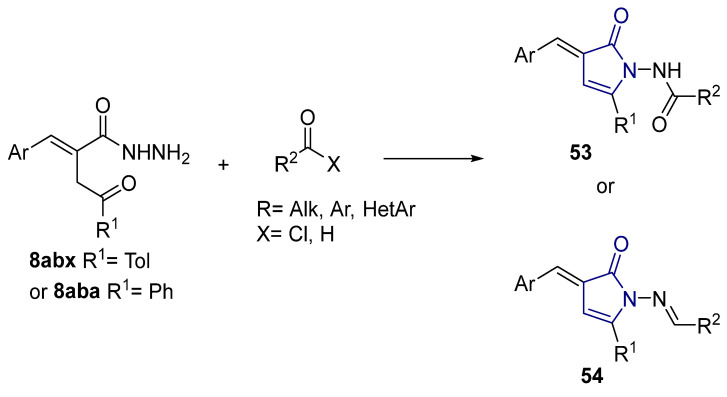
Representative scheme of pyrrolone synthesis from hydrazides.

According to the literature mentioned in Table 4, from 2019 to 2024, [27,28,101,137,140], pyrrolones **53** and **54** can be formed from the reaction of hydrazides (compounds **8aax** or **8aba**) and electrophiles (e.g., acyl chlorides or aldehydes) (Scheme 12) [27,28,101,137,140]. The reactions with acyl chlorides occurred under reflux [27,28,140] or at room temperature [28,137], and in some cases, a base [28] was used. When the reaction occurred with aldehydes [28,101,140], the reactions were performed via the catalysis of acetic acid, in ethanol, under reflux conditions. The products were generally obtained in good yields.

##### Biological Activity of Pyrrolone Derivatives

**Figure 17:**
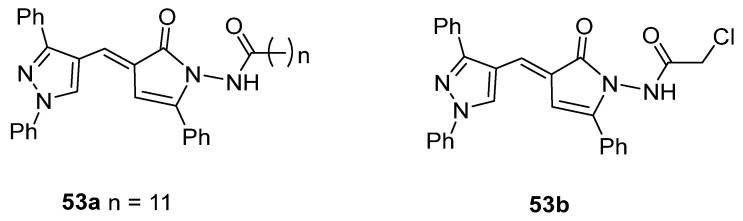
Pyrrolones with anticancer activity.

The pyrrolone derivative **53a** exhibited great in vitro anticancer activity against HCT-116 and MCF-7 cell lines, with IC_50_ = 7.49 and 8.51 µM [27], respectively (Figure 17). Also, compound **53b** showed IC_50_ = 46.3 µg/mL against HePG2 cell lines [137].

**Figure 18:**
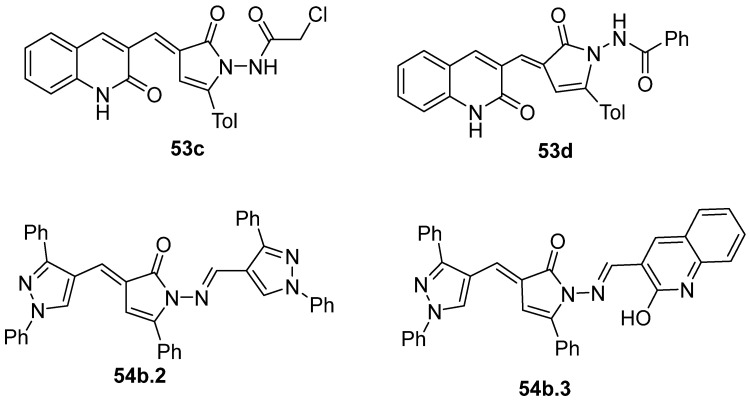
Pyrrolones with antiviral activity.

Compounds **53c** and **53d**, reported by El-Helw et al. [28], showed a high percentage of protection against the pathogenic avian influenza virus (H5N8) [28], higher than 80% of immunomodulators. Morsy et al. [101] reported on compounds **54b.2** and **54b.3**, which exhibited antiviral activity with 100% protection against Newcastle disease virus (Figure 18).

**Figure 19:**
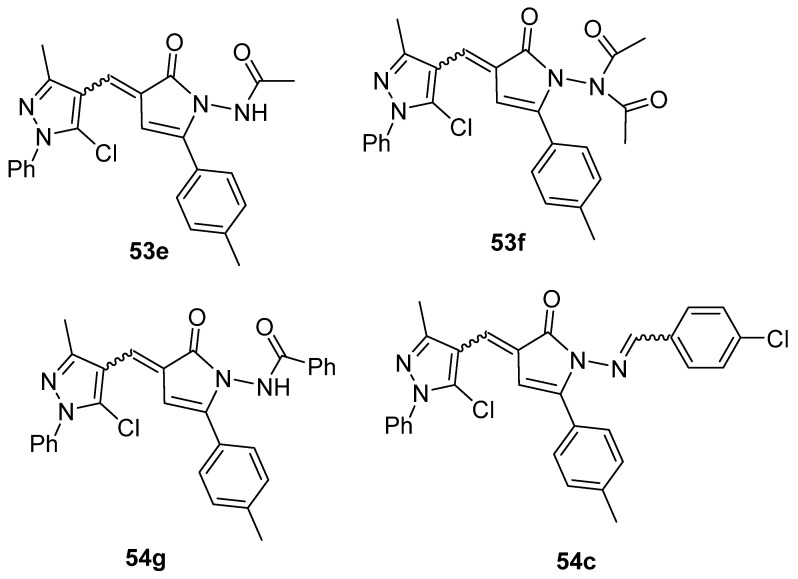
Pyrrolones with antioxidant activity.

Moreover, Youssef et al. [140] used the phosphomolybdenum method to determine the antioxidant capacity of compounds **53e**–**g** and **54c** (Figure 19). The compounds showed good to moderate antioxidant capacity, presenting 163.0 to 262.27 mg of acid ascorbic equivalents per gram (AEE/g) of dry compound.

#### 3.2.2. Pyrazoles

##### Synthesis of Pyrazoles

**Figure 20:**
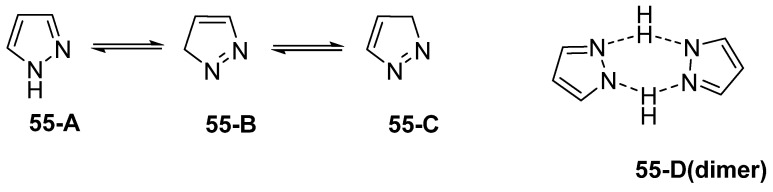
Tautomeric structures and dimer of pyrazole.

Pyrazole derivatives are five-membered *N*-heterocycle compounds with two adjacent nitrogen atoms (1,2-positions) [29]. Unsubstituted pyrazole is a planar structure with three possible tautomeric forms (**55-A**, **55-B**, and **55-C**), as represented in Figure 20**.** However, it can also exist as a dimer (**55-D**), in concentrated solution, via hydrogen bonding [142].

In the azole family, pyrazole derivatives are one of the most studied compounds, with a wide range of chemical and biological properties [29,143,144]. In clinical use, rimonabant, sildenafil, fomepizole, celecoxib, and ruxolitinib are some of the pyrazole-based drugs [142]. In the literature, pyrazoles have been described as antimicrobial [128,145,146], anti-inflammatory [147,148], and anticancer agents [149,150,151].

Hassani et al. [29] and Ríos et al. [143] compiled the works reporting the synthesis of pyrazole derivatives between 2013 and 2023 and between 2017 and 2022, respectively. Pyrazoles were obtained from the reaction between hydrazine and a carbon unit, such as 1,3-dicarbonyl, α,β-unsaturated carbonyl compounds, acetylenic ketones, or β-enaminones or similar compounds.

**Scheme 13:**
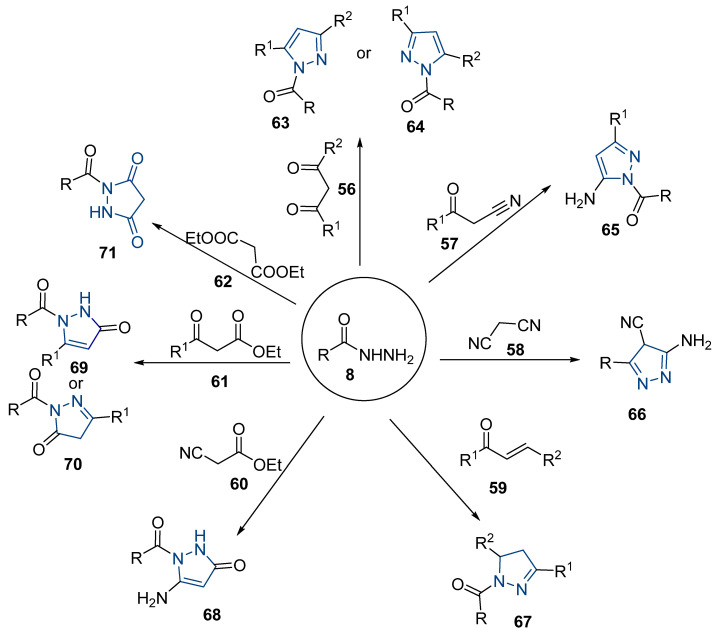
Representative scheme of pyrazoles, dihydropyrazoles, and pyrazolidine-dione synthesis from hydrazides.

Although pyrazoles are usually obtained from hydrazine, in this review, we present hydrazides as precursors of pyrazoles, dihydropyrazoles, or pyrazolidine-diones (Table 5). The synthesis of these compounds occurred between hydrazides **8** and several carbonyl/nitrile compounds as represented in Scheme 13. Pyrazoles **63** to **66** were obtained in ethanol, under reflux, in the presence or not of an organic base [80,146]. Dihydropyrazoles **67**–**70** [80,128,146] or pyrazolidine-diones **71** [80] were generated from hydrazides and carbonyl/nitrile compounds in the presence of a strong inorganic base, in ethanol or DMF, at room temperature or under reflux. The products were usually obtained with good yields [80].

**Scheme 14:**
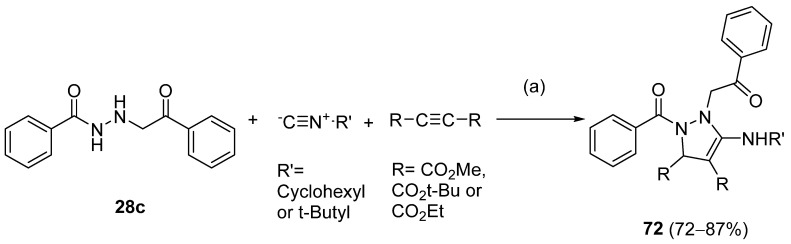
Synthesis of dihydropyrazole derivatives **48**: (a) acetone, –5 °C, 10 min, r.t., 24 h.

Also, recently, Ardakani et al. [145] reported the synthesis of dihydropyrazole **72** by the reaction of substituted hydrazide **28c** with alkyl isocyanides and dialkyl acetylenedicarboxylates at room temperature, in 72–84% yields (Scheme 14).

##### Biological Activity of Dihydropyrazole and Pyrazole Derivatives

**Figure 21:**
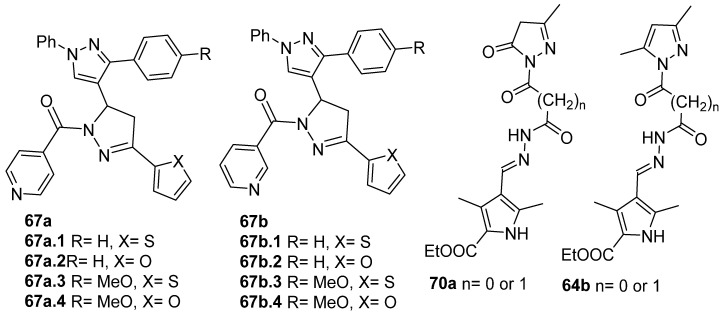
Pyrazole derivatives with antibacterial, antifungal, or anticancer activities.

Compounds **67a**,**b** (Figure 21) were screened for their in vitro antibacterial and antifungal activities. Compound **67a.2**, with an MIC = 100 µg/mL against Gram-positive *B. subtilis* and a stronger MIC = 50 µg/mL against *C. tetani*, was equipotent or more potent than the reference drugs ampicillin (MIC 250 µg/mL) and ciprofloxacin (MIC 100 µg/mL). Compound **67a.4** was more potent than ampicillin against *S. aureus* (MIC 62.5 µg/mL). In general, compounds **67a**, with isoniazid moieties, were more effective against all microorganisms than those with nicotinic hydrazide derivatives **67b** (Figure 21) [128].

Compounds **70a** and **64b** (Figure 21) demonstrated effective antibacterial activity against *Staphylococcus aureus*, *Bacillus subtilis*, *E. coli,* and *Pseudomonas aeruginosa*, with MIC values ranging from 8 to 16 µg/mL, and good cytotoxicity in vitro against two human cancer cells, HCT-116 (colon) and HL-60 (leukemia), though it was less than the standard 5-fluorouracil [146].

#### 3.2.3. Oxadiazoles

##### Synthesis of Oxadiazole Derivatives

**Figure 22:**
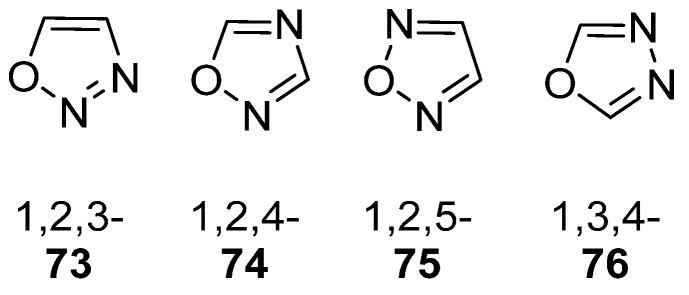
Structures of oxadiazole isomers.

Oxadiazoles are one of the most valuable five-membered heterocycles, holding one oxygen and two nitrogen atoms, with an extensive spectrum of applications [31]. From the oxadiazole isomers of 1,2,3-oxadiazole **73**, 1,2,4-oxadiazole **74**, 1,2,5-oxadiazole **75**, and 1,3,4-oxadiazole **76**, presented in Figure 22, 1,3,4-oxadiazole **76** stands among the most studied and used, due to its broad activity spectrum [152,153]. This isomer appears in some available drugs, such as Zibotentan, Furamizole, Raltegravir, and Nesapidil [83], but recently, new derivatives have been shown to have biological activities, including anticancer [82], antibacterial, antifungal [154], antimalarial [2], antileishmanial [40], antitubercular [81], antiviral [28], anti-inflammatory [41,85], antioxidant [105], and insecticidal activities [103].

Sharma et al. [31] collected and discussed the synthesis of 1,3,4-oxadiazoles in the past 15 years. The authors discussed dehydrogenative cyclization of 1,2-diacylhydrazines with phosphorus oxychloride (POCl_3_), phosphoric acid (H_3_PO_4_), and thionyl chloride (SOCl_2_); oxidative cyclization of hydrazide–hydrazones; and decarboxylative cyclization.

**Scheme 15:**
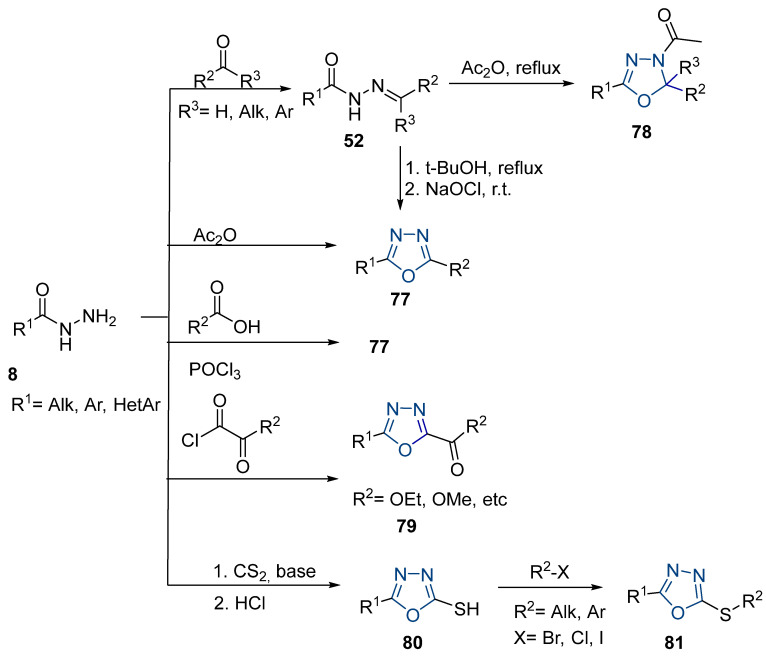
Representative scheme of 1,3,4-oxadiazole synthesis from hydrazides.

Here, we report the use of hydrazides in the synthesis of 1,3,4-oxadiazoles. According to Table 6, 1,3,4-oxadiazoles can be synthesized from the reaction between a hydrazide and carbon electrophilic reagents such as aldehydes, oxalyl chlorides, carboxylic acids, or carbon disulfide, as represented in Scheme 15. Some 2,3-dihydro-1,3,4-oxadiazol-2-yl derivatives **78** have been synthesized from hydrazides **8**, with hydrazide–hydrazones **52** as intermediates [40,82,154]. This method starts with the reaction of hydrazide and an aldehyde, and then the reaction follows in the presence of acetic anhydride under reflux. The oxadiazoles **78** obtained by this method were generally obtained in low to excellent yields. On the other hand, Paidi et al. [153] reported the synthesis of 2,5-disubstituted 1,3,4-oxadiazoles **77** via one-pot NaOCl-mediated oxidative cyclization from hydrazide–hydrazones **52**, generated in situ from hydrazides **8** and aldehydes (Scheme 15). The best conditions reported by Paidi et al. [153] included hydrazide **8** in the presence of aldehydes and *t*-BuOH, under reflux, followed by a reaction with 10–12% aqueous NaOCl at room temperature. These reaction conditions were applied to hydrazides and aldehydes with both electron-donating and electron-withdrawing groups, and the desired products **77** were obtained in moderate to excellent yields. Compounds **77** were also generated directly from **8** by reaction with acetic anhydride [80].

The reaction of hydrazides with carboxylic acids is one of the most widely used processes to produce 2,5-substituted-1,3,4-oxadiazoles **77**. The reaction normally occurs in the presence of POCl_3_ under high temperatures or reflux, yielding the products in reasonable to excellent yields [2,59,83,84,105,152].

The reactions between hydrazides and oxalyl chlorides occur at room temperature in the presence of a base or at high temperatures in the presence of POCl_3_ to produce compounds **79** [39].

Additionally, hydrazide derivatives **8** in the presence of carbon disulfide and a base (KOH, pyridine, or NaOH) are cyclized to produce different 1,3,4-oxadiazole-2-thiol derivatives **80** (Scheme 15) [28,36,37,41,61,62,63,64,86,87,103,146]. These reactions occurred generally under reflux, and the product **80** is isolated in good yield after neutralization of the reaction mixture with HCl (Table 6). Some of the compounds **80** were then alkylated with various alkyl/aryl halides to generate the new derivatives **81** (Table 6) [36,61,62,64,85,86,87].

**Scheme 16:**
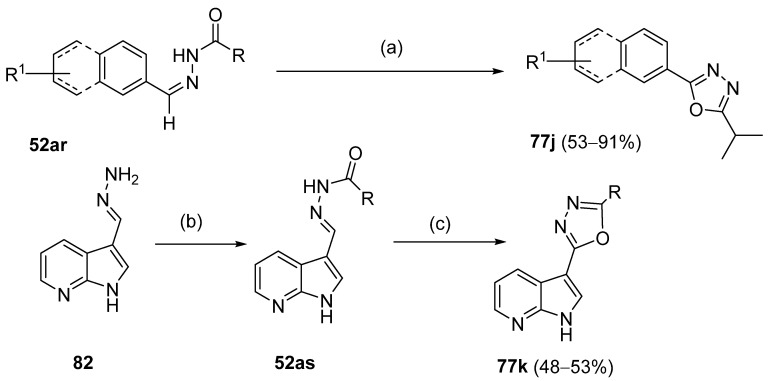
Synthesis of oxadiazole derivatives: (a) Me_2_CHCHO, p-anisolyliodide (0.1 eq), O_2_, acetone, 35 °C, 6–8 h; (b) RCOCl, Et_3_N, CH_3_CN, r.t.; (c) DMSO, I_2_, K_2_CO_3_, 110 °C, 24 h.

On the other hand, 1,3,4-oxadiazoles **77** can also be obtained in reasonable to excellent yields by the iodine-mediated synthesis approach [155,156]. Chauhan et al. [155] reported the synthesis of oxadiazoles **77j** from **52ar** in the presence of isobutyraldehyde and *p*-anisolyl iodide under auto-oxidation conditions in the presence of molecular oxygen. The 2,5-disubstituted 1,3,4-oxadiazole **77k** was obtained from substituted carbohydrazides **52as** by the iodine-mediated synthesis approach represented in Scheme 16 [156].

**Scheme 17:**
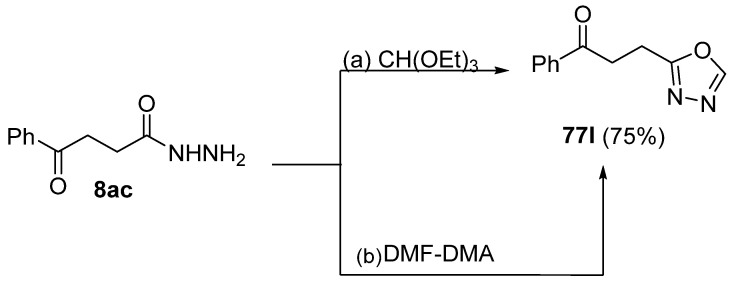
Synthesis of 2-substituted 1,3,4-oxadiazole **77l**: (a) CH(OEt)_3_, reflux, 15–18 h; (b) DMF–DMA.

Moreover, Abu-Hashem et al. [80] reported the synthesis of 1,3,4-oxadiazoles **77l** (Scheme 17) starting from hydrazide **8ac** in the presence of triethyl orthoformate or dimethylformamide dimethyl acetal [80].

**Scheme 18:**
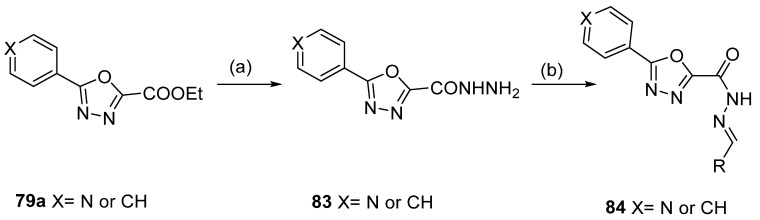
Synthesis of 2,5-disubstituted 1,3,4-oxadiazole derivatives: (a) H_2_NNH_2_, EtOH, reflux; (b) RCOH (R = Ar, HetAr), EtOH, CH_3_COOH (cat.), reflux.

1,3,4-oxadiazoles **79a** were used to generate new derivatives **83** and **84**. Compounds **79a** reacted with hydrazine to generate the corresponding hydrazide **83**, which was converted to **84** by reaction with an aldehyde under acid catalysis [81] (Scheme 18).

**Scheme 19:**
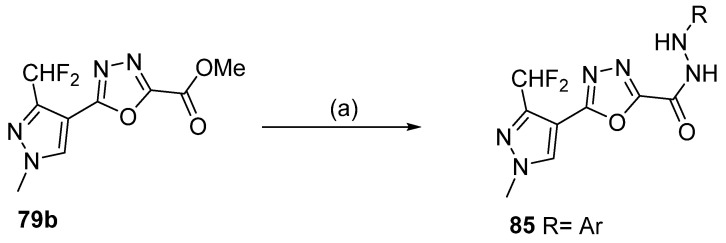
Synthesis of 1,3,4-oxadiazole derivative **85**: (a) 1-butyl-3-methyl imidazolium chloride, RNHNH_2_, Ar, 100 °C.

Moreover, 1,3,4-oxadiazole derivatives **85** were obtained by reacting derivative **79b** with aryl hydrazines in an ionic liquid at 100 °C (Scheme 19) [39].

##### Biological Activity of Oxadiazole Derivatives

**Figure 23:**
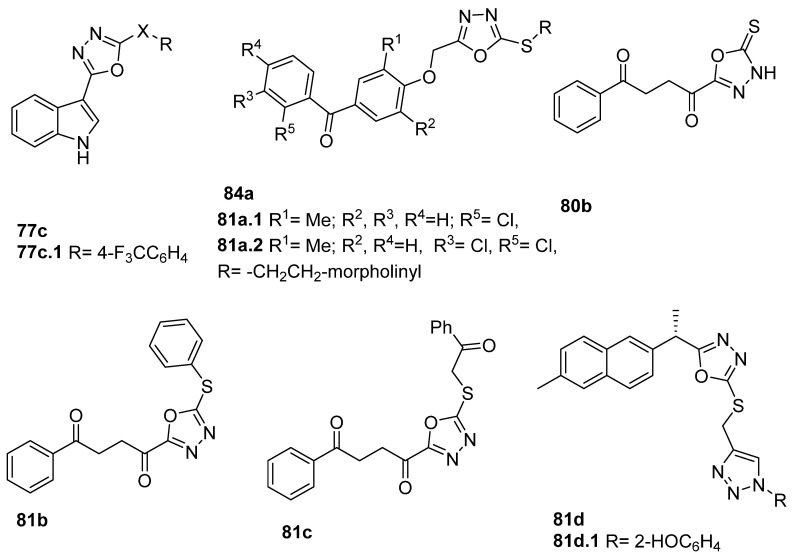
1,3,4-oxadiazoles with anticancer activity.

Hamdy et al. [84] reported the new series of 2-(1H-indol-3-yl)-5-substituted-1,3,4-oxadiazoles **77c** as inhibitors of the Bcl-2 anti-apoptotic gatekeeper protein. Compound **77c.1** (Figure 23) exhibited potent anticancer activity with IC_50_ = 0.52, 0.88, and 0.73 µM against MDA-MB-231, HeLa 2, and KG1a3 (Bcl-2-expressing) cell lines, respectively. Moreover, it showed no inhibitory effects in the Bcl-2-negative Jurkat cell line.

Derivatives **81a.1** and **81a.2** (Figure 23) showed excellent activities against the MCF-7 cell line (breast cancer), with IC_50_ = 127.0 and 126.7 µg/mL, respectively, and against KB (oral cancer) cell lines, with IC_50_ = 113.8 and 112.6 µg/mL. Furthermore, these compounds revealed no toxicity against the normal cell line L929 at higher concentrations with IC_50_ > 201 μg/mL [86].

Oxadiazole derivatives **80b**, **81b**, and **81c** (Figure 23) presented antiproliferative activities against MCF-7 breast cancer cells with IC_50_ values between 8.2 and 8.8 µM, which were lower compared to doxorubicin (10.3 µM), and against hepatocellular HepG2 cells with IC_50_ values between 26.9 and 29.8 µM, which were quite similar compared to doxorubicin (28.5 µM) [64].

Among the derivatives **81d**, oxadiazole **81d.1** showed the highest activity against MCF-7 and HepG2 cancer cell lines with IC_50_ = 2.13 and 1.63 µg/mL, respectively. In addition, compound **81d.1** inhibited EGFR kinase with IC_50_ = 0.41 µM [36].

**Figure 24:**
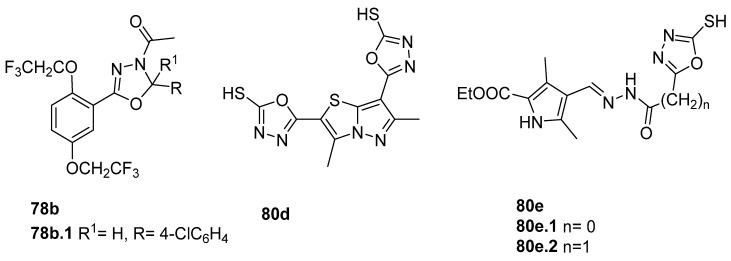
1,3,4-oxadiazole-2-thiol derivatives with anticancer and/or antibacterial activities.

Shankara et al. [82] evaluated the anticancer activity of compounds **78b** (Figure 24), and among them, compound **78b.1** was the most potent with an IC_50_ = 8.14 µM against the LN229 Glioblastoma cell line. Also, derivative **80d** (Figure 24) showed good activities for HepG2 and HCT-116, with IC_50_ = 6.9 and 13.6 µg/mL, respectively [37]. Rawat et al. [146] reported on compounds **80e.1** and **80e.2** (Figure 24), showing good cytotoxicity against HCT-116 and HL-60 (leukemia) cell lines, with the growth inhibition percentage being superior to 70% at 5 µg/mL, but less than the standard 5-fluorouracil (a growth inhibition percentage superior to 85% at the same concentration). Compounds **80e** also showed good antibacterial activity with MICs between 2 and 8 µg/mL against Gram-positive bacteria (*Staphylococcus aureus*, Bacillus subtilis) and Gram-negative bacteria (*E. coli*, *Pseudomonas aeruginosa*) [146].

**Figure 25:**
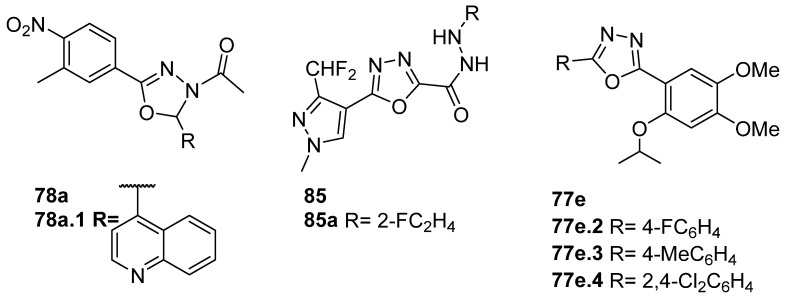
1,3,4-oxadiazoles as antibacterial and antifungal agents.

Compound **78a.1** (Figure 25) showed promising activity against *Staphylococcus epidermidis* with an MIC = 0.48 µg/mL, as well as low cytotoxicity against the L929 normal cell line [154].

Long et al. [39] designed, synthesized, and evaluated oxadiazole derivatives **85** (Figure 25) for their antifungal, antioomycete, and antibacterial activities. Compound **85a** showed the best in vitro antifungal activity against *Gibberella zeae* and antioomycete activity against *Phytophora infestins*, with EC_50_ = 0.47 µg/mL and 3.92 µg/mL, respectively. In the in vivo study against corn scab, compound **60a** showed protective and curative activities of 90.2 and 86.3% at 200 µg/mL, which were comparable to those of fungicides boscalid and fluopyram. These 1,3,4-oxadiazole-tailored pyrazole compounds with hydrazide functions in the middle as a linker are potential agricultural fungicides for controlling fungal diseases.

The 2,5-disubstituted 1,3,4-oxadiazoles **77e** (Figure 25) were evaluated for their in vitro antibacterial activity. Compound **77e.2** exhibited the best broad-spectrum antibacterial and antifungal activity, with MIC = 15.62, 7.81, 3.9, and 31.25, 62.5 µg/mL against *E. coli*, *S. typhi*, *B. subtilis*, *B. megaterium*, and *A. niger*, respectively [83].

**Figure 26:**
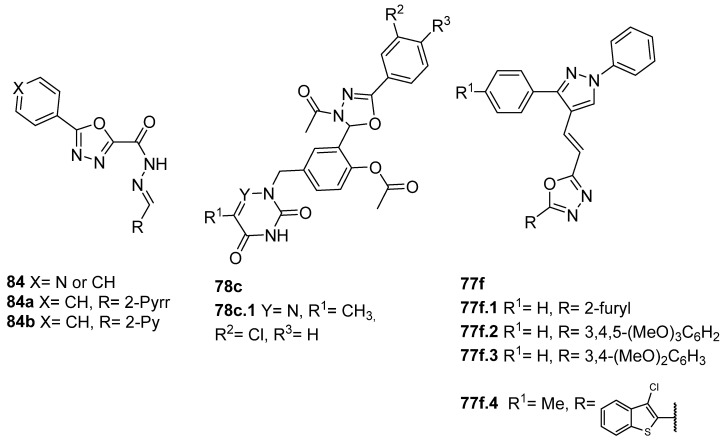
1,3,4-oxadiazoles with antimycobacterial and antiparasitic activities.

Derivatives **84** (Figure 26) were evaluated for their in vitro antimycobacterial activity against the *M. tuberculosis* H37Ra-attenuated strain, H37Rv virulent strain, and several resistant strains. From the 5-phenyl-substituted oxadiazole subseries, derivatives **84a** and **84b** presented an MIC = 4 µM against pyrazinamide-resistant strains. Moreover, these compounds exhibited selectivity for mycobacteria and low cytotoxicity against human SH-SY5Y cells (CC_50_ = 50 and 100 µM for **84a** and **84b**, respectively) [81].

Also, *N*^3^-acetyl-1,3,4-oxadiazoline derivatives **78c** (Figure 26) were screened against *Leishmania donovani*, and compound **78c.1** exhibited an antileishmanial activity with IC_50_ = 8.98 µM on *L. donovani* intramacrophage amastigotes [40].

Moreover, Verma et al. [2] synthesized the hybrid compounds **77f** and evaluated their activity against *P. falciparum* 3D7 (chloroquine-sensitive) and RKL 9 (chloroquine-resistant) strains. Among the evaluated compounds **77f** (Figure 26), compound **77f.1** exhibited the best activity with an IC_50_ = 0.25 µg/mL against the 3D7 (chloroquine-sensitive) strain and 0.86 µg/mL against the RKL 9 (chloroquine-resistant) strain of *P. falciparum*. Moreover, the antileishmanial activity of compounds **77f** against *L. donovani promastigotes* was also evaluated. Compounds **77f.2**, **77f.3**, and **77f.4** exhibited IC_50_ = 33.3, 40.1, and 19.0 µg/mL, respectively. The same compounds (**77f.2**, **77f.3**, and **77f.4**) also had effects on amastigote infectivity with IC_50_ = 44.2, 66.8, and 73.1 µg/mL, respectively. Among the tested compounds, the most promising were **77f.1** and **77f.4** for their good antimalarial and antileishmanial activity, respectively; hence, their cytotoxicity was studied, as well as their safety profile.

**Figure 27:**
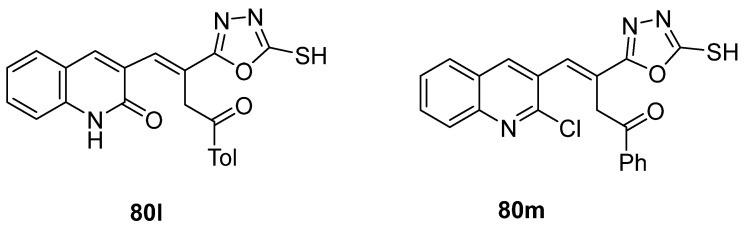
1,3,4-oxadiazole-2-thiol derivatives with antiviral (**80l**) or insecticidal activity (**80m**).

El-Helw et al. [28] reported compound **80l** (Figure 27) as an immunomodulator against the highly pathogenic avian influenza virus (H5N8), with the high potency of 100% protection, and Ramadan et al. [103] reported compound **80m** (Figure 27) as an insecticide with low LC_50_ = 9.67 and 1.07 mg/mL against lab and field strains of the third larval instar of *Culex pipiens.*

**Figure 28:**
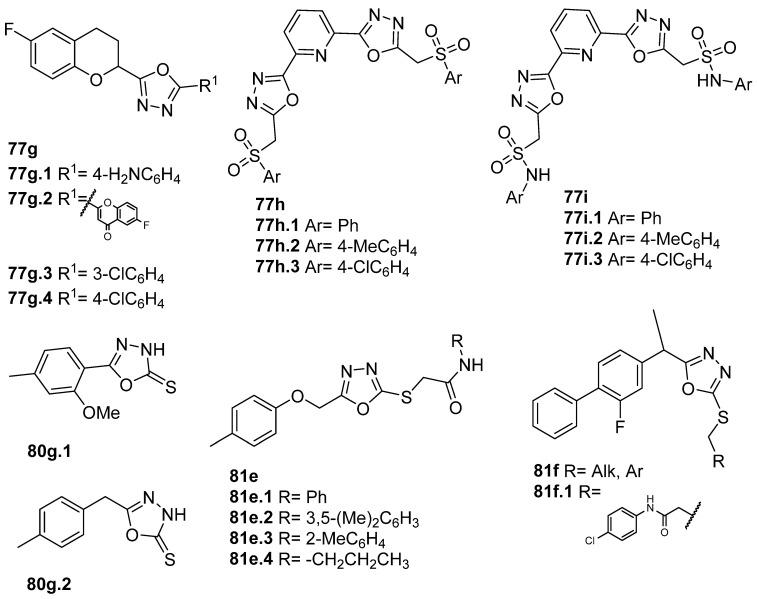
1,3,4-oxadiazole derivatives as potential anti-inflammatory and antioxidant agents.

New derivatives of novel 2,5-disubstituted 1,3,4-oxadiazole (Figure 28) were also synthesized as potential anti-inflammatory and antioxidant agents. Kashid et al. [59] reported compounds **77g** (Figure 28) with great anti-inflammatory and antioxidant activities, of which compounds **77g.1**, **77g.2**, and **77g.3** showed better anti-inflammatory activities with IC_50_ = 45.69, 58.54, and 56.70 µM, respectively, compared to the standard drug diclofenac sodium that presents an IC_50_ = 90.21 µM. According to the DPPH assay, compound **77g.4** exhibited good antioxidant activity with IC_50_ = 17.15 µM, which was better than the reference antioxidant ascorbic acid (IC_50_ = 44.18 µM). Also, a molecular docking study showed that these compounds can recognize the active site and accomplish significant bonded and non-bonded interactions with main residues in the anti-inflammatory target cyclooxygenase-2 (COX-2) [59]. Gunthanakkala et al. [105] reported compounds **77h** and **77i** (Figure 28) as potential antioxidants with IC_50_ values between 32.95 and 121.12, 29.90 and 117.73, and 31.34 and 106.42 µg/mL, for DPPH, NO, and H_2_O_2_ assays, respectively. Among them, compound **77h.2** stood out with IC_50_ values = 32.95, 31.64, and 32.42 μg/mL, and compound **77i.2** with IC_50_ values = 32.01, 29.90, and 31.34 µg/mL for DPPH, NO, and H_2_O_2_ assays, respectively.

Some oxadiazole-2-thiol derivatives **80g** (Figure 28) also presented anti-inflammatory activity as inhibitors of COX or lipoxygenase (LOX) enzymes [41,85]. Munir et al. [41] identified derivatives **80g** with good in vitro cyclooxygenase inhibition activity, with IC_50_ values ranging from 31.5 to 39.5 µM for COX-2 and from 43.91 to 27.55 µM for COX-1. On the other hand, Bashir et al. [85] identified oxadiazoles **81e.1**–**4**, which showed good LOX inhibitory activities with IC_50_ values of 21.5, 29.1, 31.3, and 24.3 µM, respectively.

Rana et al. [61] reported new derivatives **81f** (Figure 28) incorporating the flurbiprofen moiety. Compound **81f.1** showed the highest anti-inflammatory activity of the series, displaying 74.16% activity at 200 µg/mL, which is slightly lower than standard ibuprofen (84.31% activity). The same compound also showed antioxidant activity in the DPPH assay, with an IC_50_ = 25.35 µg/mL, while for ascorbic acid, the IC_50_ value was 6.13 µg/mL.

**Figure 29:**
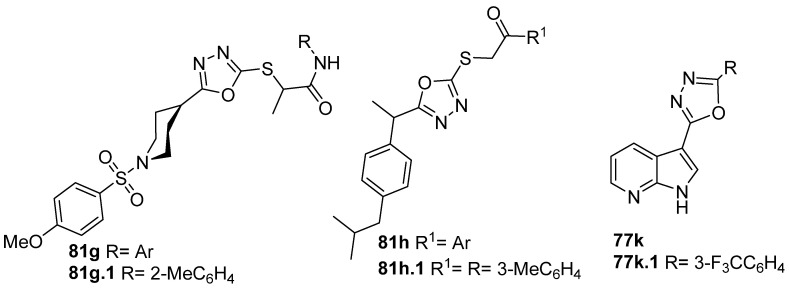
Oxadiazole derivatives as α-glucosidase inhibitors.

Furthermore, both derivatives **81g** and **81h** (Figure 29) showed good inhibition against α-glucosidase. Compound **81g.1** had the inhibition potential of 72.13% at 500 µM, which was higher than that of the standard drug acarbose (65.73% at 500 µM) [87]. Additionally, Daud et al. [62] identified compound **81h.1** with IC_50_ = 56.01 µM as more active than acarbose, the standard drug, which presents an IC_50_ = 375.82 µM, in the same assay. Compound **77k.1** also showed good α-glucosidase inhibition activity with an IC_50_ = 460 µM [156].

#### 3.2.4. Thiazoles and Thiadiazoles

##### Synthesis of Thiazole and Thiadiazole Derivatives

**Scheme 20:**
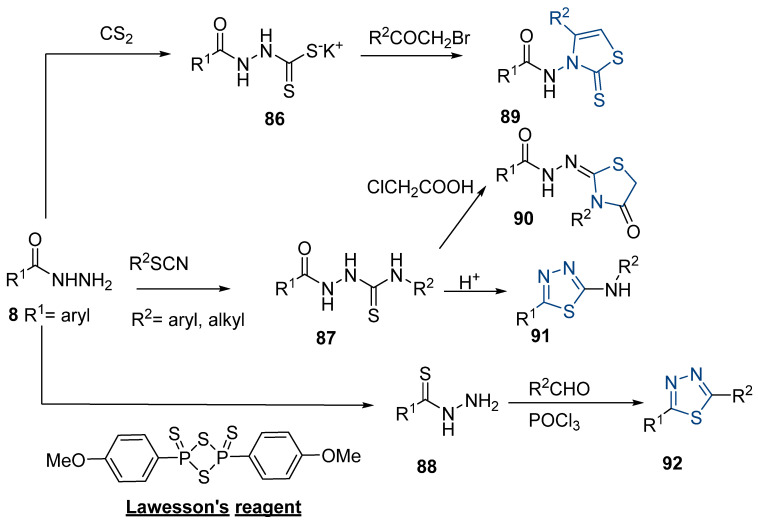
General approaches to 1,3-thiazole and 1,3,4-thiadiazole synthesis from hydrazides.

Thiazole and thiadiazole are five-membered N,S- and N,N,S-heterocycles with important biological applications that foster the search for new derivatives. Recently, Babalola et al. [157] and Ahmad et al. [158] collected and discussed recent synthetic methodologies and the biological activity of thiadiazoles. Babalola et al. [157] discussed the synthesis of thiadiazoles over the last 10 years using heterogeneous catalysts, microwave-assisted synthesis, ultrasound-aided techniques, solvent-free synthesis, or complex catalyzed reactions; Ahmad et al. [158] discussed the synthesis of thiadiazoles, since 2008, from hydrazides, thiosemicarbazide, acylhydrazines, thioacylhydrazone, dithiocarbazates, and isothiocyanate. Scheme 20 presents the general approaches to obtain these heterocycles from hydrazides **8**, and Table 7 describes the reaction conditions to obtain the different derivatives from the reactions between hydrazides and carbon disulfide, isothiocyanate reagents, or Lawesson’s reagent.

Tolan et al. [64] reported the synthesis of compounds **89** in a two-step approach (Scheme 20). The reaction of hydrazide **8** with carbon disulfide produced intermediary **86**, which reacted with an acyl bromide reagent in ethanol and was refluxed to generate the thiazole ring of derivative **89**. Abumelha et al. [44] synthesized thiazole derivatives **90** (Scheme 20). The synthetic approach involved the conversion of hydrazide **8** into intermediate **87**, by reaction of **8** with isothiocyanate, under heating. The reaction of intermediate **87** with chloroacetic acid promoted the formation of thiazole ring **90**. Moreover, intermediary **87** was converted to 1,3,4-thiadiazoles **91** by treatment with sulfuric acid under reflux [94,159].

Hydrazides **8**, in the presence of Lawesson’s reagent, generated the corresponding thio-derivatives **88**, which generated 1,3,4-thiadiazole derivatives **92** in the presence of phosphoryl chloride and an aldehyde, under heating (Scheme 20) [93].

**Scheme 21:**
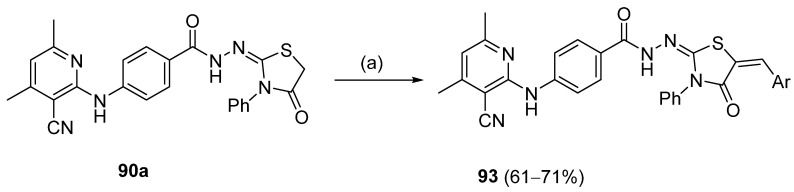
Synthesis of thiazole derivative **93**: (a) ArCHO, CH_3_COOH/AcONa, reflux, 4 h.

Abumelha et al. [44] synthesized thiazole derivatives **93** as precursors of antioxidant agents. Compound **90a** was converted to the hybrids **93** by reaction with aldehydes under reflux, in an acidic medium. Products **93** were obtained in good to moderate yields (Scheme 21).

**Scheme 22:**
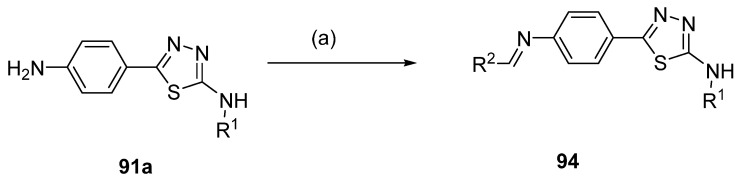
Synthesis of thiadiazole derivative **94:** (a) R^2^CHO, MeOH, reflux.

Compounds **94** (Scheme 22) were yielded from thiadiazole derivative **91a** and aldehydes under reflux conditions, in methanol [94].

##### Biological Activity of Thiazole and Thiadiazole Derivatives

**Figure 30:**
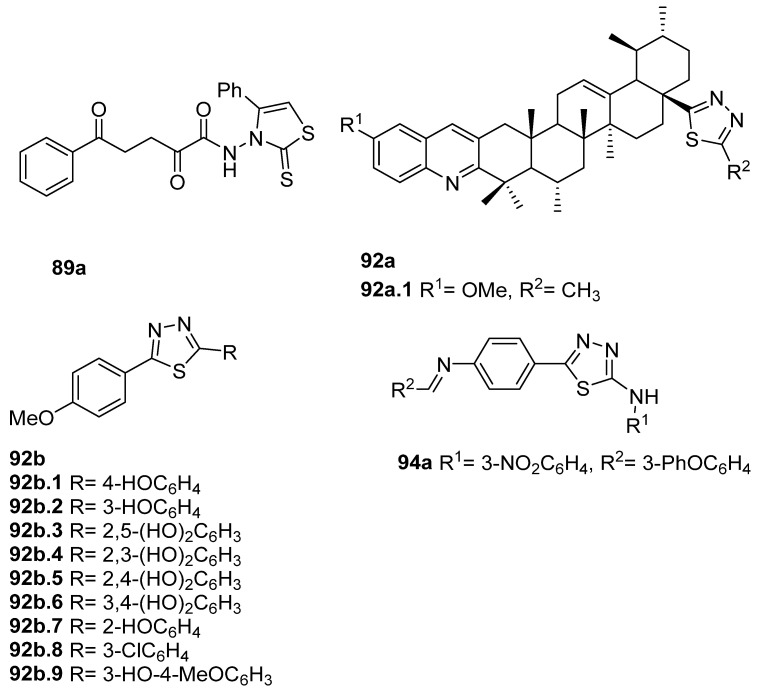
Thiazole and 1,3,4-thiadiazole derivatives with anticancer and antimycobacterial activity.

Compound **89a** (Figure 30) was evaluated for anticancer activity and showed good antiproliferative activities against MCF-7 breast cancer cells and against hepatocellular HepG2 cells with IC_50_ = 8.0 and 28.2 µM, respectively. These IC_50_ values are better or similar to those of doxorubicin, which has IC_50_ = 10.3 and 28.5 µM, for the same cell lines [64].

Compounds with structure **92a** (Figure 30) were screened against cancer cell lines MDA-MB-231 and HeLa. Compound **92a.1** (Figure 30) exhibited good anticancer activity with IC_50_ = 15.75 and 12.82 µM against cancer cell lines MDA-MB-231 and HeLa, respectively, although it had a lower activity than the positive control etoposide [108]. Taha et al. [93] reported the 2,5-disubstituted thiadiazoles **92b** (Figure 30) as potent *β*-glucuronidase inhibitors presenting IC_50_ values between 6.74 and 52.36 µM, revealing higher or equivalent activity to the standard *D*-saccharic acid-1,4-lactone (IC_50_ = 48.4 µM). Among these, compound **92b.5** was the most potent, with IC_50_ = 6.74 μM.

On the other hand, thiazoles **94** (Figure 30) were screened against *Mycobacterium tuberculosis* H_37_Rv, and compound **94a** was the most potent with an inhibitory activity of 80% at 6.25 µg/mL [94].

#### 3.2.5. Triazoles

##### Synthesis of Triazole Derivatives

**Figure 31:**
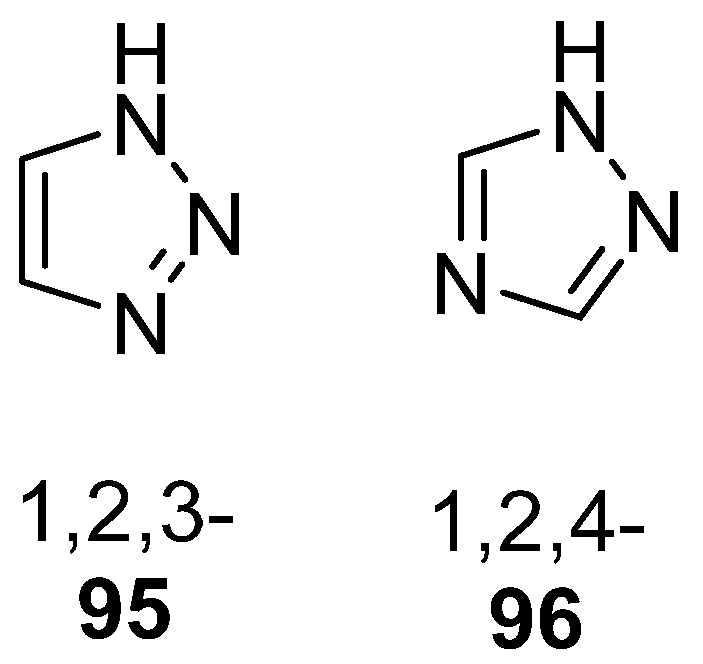
Isomeric forms of the triazole ring.

Triazole, also known as pyrrodiazole, is a five-membered heterocyclic ring system containing three nitrogen atoms, existing in two isomeric forms, 1,2,3- **95** or 1,2,4-triazoles **96** (Figure 31) [160]. Both isomers present a wide range of pharmacological activities.

In a recent review, Hassani et al. [161] reported the advances in the synthesis of triazole derivatives. The authors presented multiple methods to obtain 1,2,3- or 1,2,4-triazoles, including metal-free and metal-catalyzed reactions. Among them are the cycloaddition of azides and terminal alkynes; the reaction between two nitriles and hydroxylamine hydrochloride; the reaction of formamide reagents and hydrazide; the reaction of acylhydrazines with carbon disulfide, followed by the reaction with hydrazine monohydrate; and others [161]. Ren et al. [162] recently reported a different approach for the synthesis of 1,2,3-triazoles, involving an iodine-mediated condensation–cyclization reaction from α-azido acetophenones and *p*-toluenesulfonyl hydrazide. Moreover, Clark et al. [163] developed the synthesis of substituted 1,2,3-triazoles from α-ketoacetals, tosyl hydrazide, and a primary amine. On the other hand, Patterson et al. [164] presented the synthesis of 1,2,3-triazoles from tosylhydrazide, aldehydes, and a primary amine, as an alternative to azides.

**Scheme 23:**
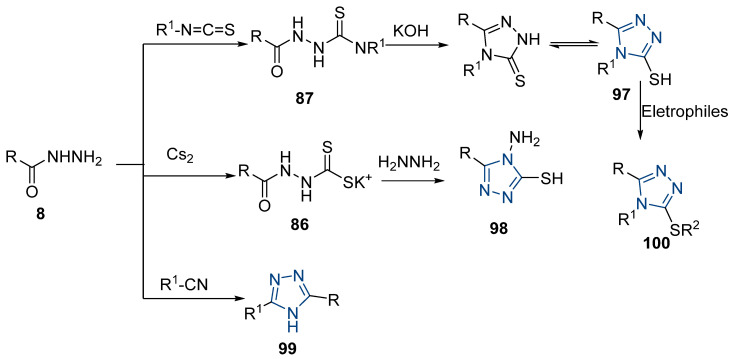
Representative scheme of 1,2,4-triazoles from hydrazides.

Here, in this review, we present the synthesis of 1,2,4-triazole (Table 8), in which hydrazides are often combined with thiocyanate or isothiocyanates, carbon disulfide, or nitrile derivatives (Scheme 23). Hydrazides **8**, in the presence of isothiocyanates and under reflux conditions, generate the intermediates **87** (in a neutral or acidic medium), which cyclize in a basic medium under reflux, to give **97** [35,42,80,88,89,91,165]. Several derivatives of **100** were obtained by the condensation of **97** with electrophiles [42,88,89,90,91]. The reaction with carbon disulfide took place in a basic medium with reflux, followed by cyclization with hydrazine hydrate to obtain compounds **98** [41]. Reflux or high temperatures are also used when nitrile derivatives are used as reagents to obtain compounds **99** [166,167]. The products **97**, **98**, and **99** were typically isolated in good to excellent yields. The experimental conditions for the synthesis of 1,2,4-triazole-3-thione derivatives are presented in Table 8. The 1,2,4-triazole-3-thione compounds were sometimes just intermediates to obtain the compounds **100** or others with potential biological activity [110].

##### Biological Activity of Triazole Derivatives

According to the literature [35,41,42,64,80,88,89,90,91], 1,2,4-triazole derivatives showed enzymatic inhibition (α-glucosidase, 15-lipooxigenase, acetylcholinesterase, and butyrylcholinesterase enzymes), as well as anticancer or anti-inflammatory activity.

**Figure 32:**
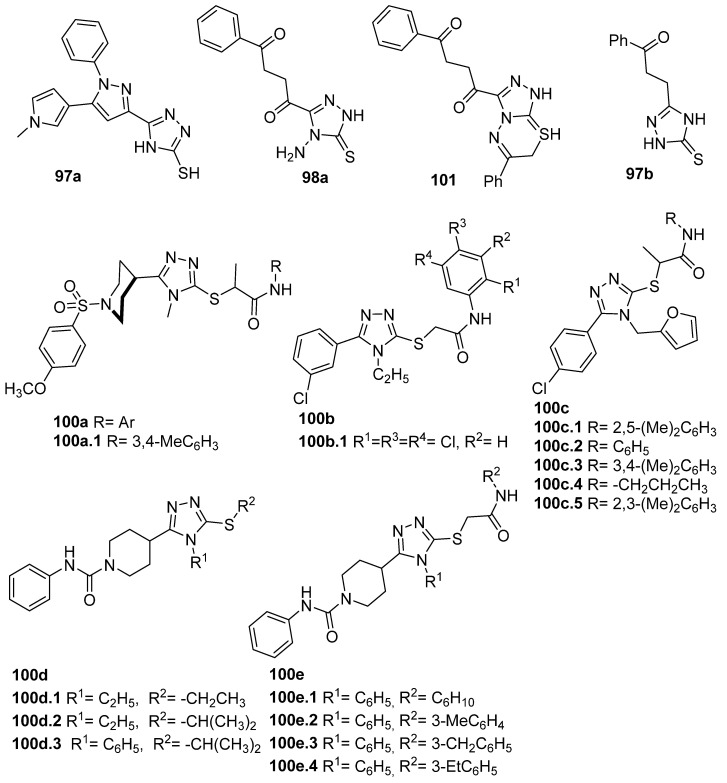
1,2,4-triazole derivatives with anticancer and enzymatic (α-glucosidase, acetylcholinesterase, butyrylcholinesterase, and 15-lipoxygenase) inhibition activity.

Derivative **97a** (Figure 32) was tested against the human colon carcinoma cancer cell line HCT-116 and showed moderate cytotoxic effects with IC_50_ = 12.05 µg/mL [35]. Both compounds **98a** and **101** (Figure 32) presented antiproliferative activities against MCF-7 breast cancer cells with IC_50_ values of 8.2 and 9.2 µM, respectively, which were lower than the IC_50_ of the reference drug doxorubicin (10.3 µM). They were also active against hepatocellular HepG2 cells with IC_50_ values of 33.7 and 30.8 µM, which were quite similar compared to doxorubicin (28.5 µM) [64]. Moreover, Abu-Hashem et al. [80] evaluated compound **97b** against human gastric carcinoma (MGC-803), nasopharyngeal carcinoma (CNE2), oral carcinoma (KB), and breast adenocarcinoma (MCF-7) cell lines. The compound showed IC_50_ values in the range of 12.8 to 14.2 µM.

Virk et al. [42] and Riaz et al. [88] evaluated the biological potential of compounds **100a** (Figure 32) against AChE. Derivative **100a.1** showed good inhibition against α-glucosidase (IC_50_ = 27.52 mM) compared to acarbose (IC_50_ = 375.82 mM) and lower inhibition against AChE (IC_50_ = 407.24 mM) in comparison with the standard drug eserine (IC_50_ = 0.19 mM) [42]. On the other hand, compound **100b.1** showed activity against AChE and BChE with IC_50_ values of 5.41 and 7.52 µM, respectively [88]. Compounds **100c**–**e** (Figure 32) were tested as potential lipoxygenase inhibitors. Among these [89], compounds **100c.1**–**5** demonstrated good activity as inhibitors of 15-lipoxygenase with IC_50_ values of 17.43, 19.35, 23.59, 26.35, and 27.53 µM, respectively. The 1,2,4-triazole thioethers **100d.1**–**3** also showed very good inhibitory profiles against the same enzyme, with IC_50_ values ranging from 12.52 to 35.64 µM [90].

Muzaffar et al. [91] reported on derivatives **100e.1**–**4** (Figure 32), which displayed inhibitory potential against the 15-lipoxygenase enzyme with IC_50_ values between 9.25 and 21.82 µM.

**Figure 33:**
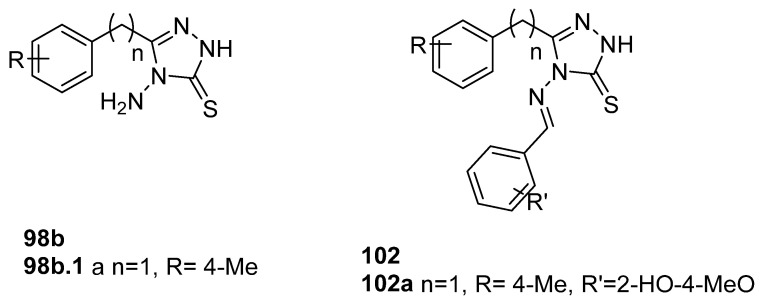
1,2,4-triazole derivatives with anti-inflammatory activity.

Munir et al. [41] obtained 1,2,4-triazole derivatives **98b** and **102** (Figure 33), and compounds **98b.1** and **102a** displayed excellent and good activity for the COX-2 isozyme with IC_50_ values of 1.76 and 23.47 µM, respectively. Other compounds **102** of this series showed COX-2 inhibition in the range of 12.56–26.58 µM. In vivo anti-inflammatory studies, by using the carrageenan-induced paw edema test, showed that after 5 h, the maximum percentage inhibition was 29.4% for compound **98b.1** and 17.6% for compound **102a** [41].

## 4. Miscellany

In the literature, hydrazides are also mentioned as reagents to generate triazine or triazepine rings [80], coating agents of nanoparticles [168], or even ligands for complexes [169,170].

**Scheme 24:**
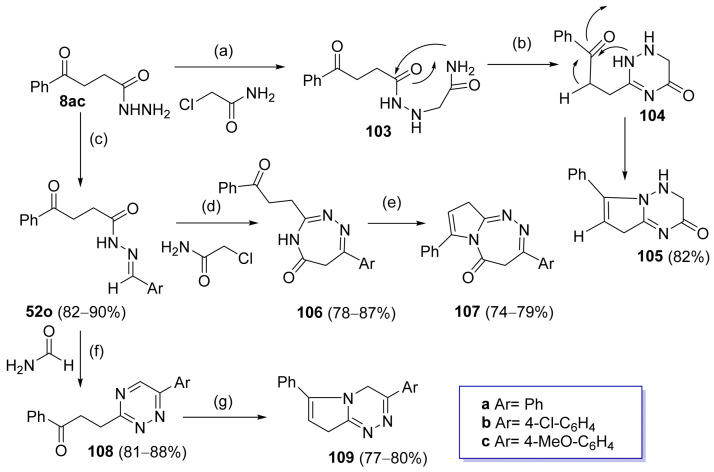
Synthesis of other heterocyclic rings from hydrazides: (a) ClCH_2_CONH_2_, DMF, reflux, 24–48 h; (b) DMF, K_2_CO_3,_ reflux, 13–16 h; (c) ArCHO, EtOH, piperidine (cat.), reflux, 5–8 h; (d) H_2_NCOCH_2_Cl, DMF; (e) DMF, K_2_CO_3_, reflux, 17–20 h; (f) H_2_NCOH, DMF, reflux, 9–24 h; (g) DMF, K_2_CO_3_, reflux, 15–18 h.

Abu-Hashem et al. [80] synthesized triazine or triazepine derivatives from hydrazide **8ac** (Scheme 24). Intermediate **103** was synthesized from hydrazide **8ac** and chloroacetamide under reflux. It was converted to **104** by reflux under basic medium. The latter further suffered an intramolecular cyclization, generating pyrrolotriazinones **105**. This hydrazide was also the starting material of hydrazide–hydrazone derivatives **52o**, which were used to obtain new compounds such as 1,2,4-triazepinones **106**, pyrrolotriazepinones **107**, 1,2,4-triazines **108**, and pyrrolotriazines **109**. Derivatives **106** were obtained from intermediate **52o** in the presence of chloroacetamide, which can undergo a posterior cyclization in the presence of a base and reflux to form compound **107.** Compound **52o** in the presence of formamide and reflux generated 1,2,4-triazines **108**, which under reflux led to the formation of pyrrolotriazines **109.** Compounds **107a**–**c**, **106a**–**c**, **109a**–**c**, and **105** displayed activities against human gastric carcinoma (MGC-803), nasopharyngeal carcinoma (CNE2), oral carcinoma (KB), and breast adenocarcinoma (MCF-7) lines with IC_50_ values ranging from 11.1 to 14.2 µM.

**Figure 34:**
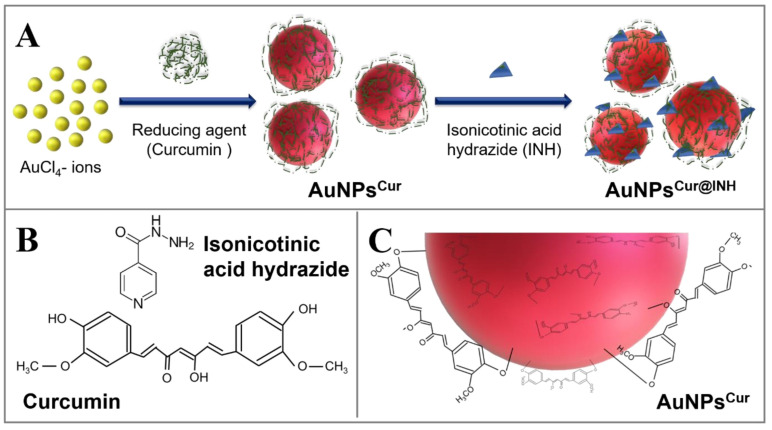
Representation of the synthesis of curcumin-coated gold nanoparticles with surface modification with isonicotinic acid hydrazide. (**A**) Schematic representation of the formation of curcumin (Cur) coated AuNPs^Cur^, and their sequential surface modification with isonicotinic acid hydrazide (INH) to develop AuNPs^Cur@INH^; (**B**) The chemical structures of curcumin and isonicotinic acid hydrazide (INH); (**C**) The phenolic and enolic hydroxide groups of curcumin are involved in the reduction of AuCl_4_^−^ ions, and orientation of oxidized moieties of curcumin on the surface of AuNPs. Reprinted with permission from Ref. [168] Copyright 2020 Elsevier.

Umapathi et al. [168] prepared gold nanoparticles with a surface corona of curcumin and isonicotinic acid hydrazide for improved anticancer activity, since gold nanoparticles can carry and stabilize these molecules. The isonicotinic acid hydrazide was used due to its biological importance. The resulting nanoparticles with the isonicotinic hydrazide at a 5 ppm concentration showed good anticancer activity towards human lung squamous carcinoma (LK-2) through ROS generation (Figure 34).

**Figure 35:**
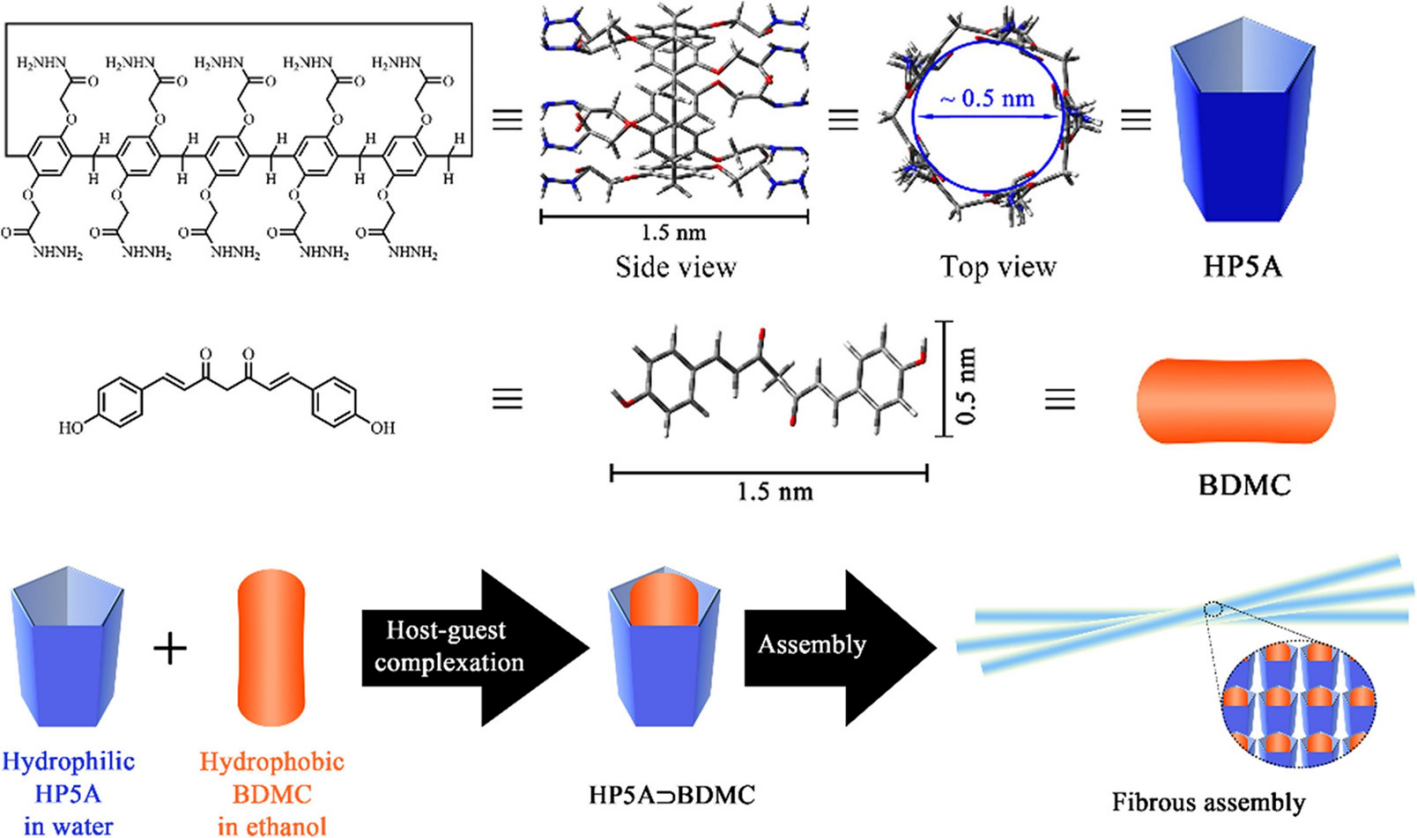
Representation of hydrazide–pillar[5]arene (HP5A), the complex of these with bisdemethoxycurcumin (BDMC), and the self-assembly into fibers. Reprinted with permission from Ref. [169] Copyright 2021 Elsevier.

To improve the water solubility and stability of bisdemethoxycurcumin (BDMC), Guo et al. [169] obtained pillar[5]arene complexes of this compound (Figure 35). They synthesized hydrazide–pillar[5]arene (HP5A) and the complex of the two (BDMC and HP5A) self-assembled into fibers. Regarding the IC_50_ of free BDMC (IC_50_ = 50.6 µg/mL) and in complex (IC_50_ = 32.4 µg/mL), this complex showed greater antiproliferative activity in vitro against hepatocellular carcinoma HepG2 cells and, at the same time, reduced the undesirable side effects on normal cells.

**Figure 36:**
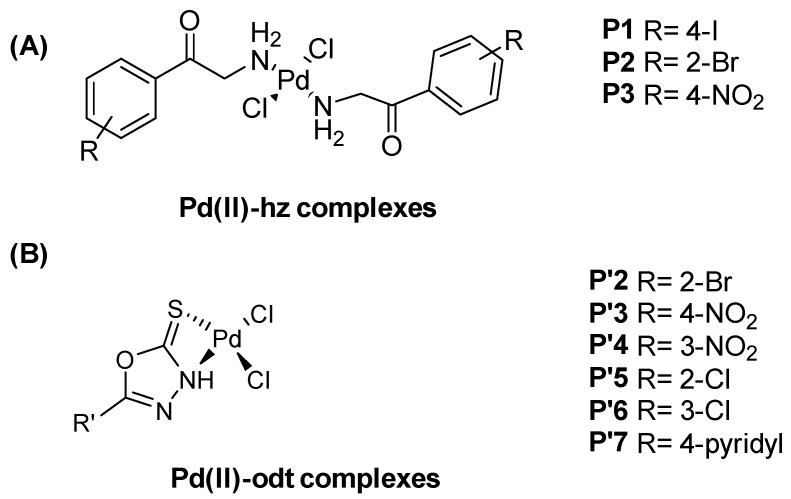
(**A**) New Pd(II)–hydrazide (Pd(II)-hz) complexes (**P1**–**P3**) and (**B**) new Pd(II)–oxadiazole (Pd(II)-odt) complexes (**P′2**–**P′7**).

In 2022, new 1,3,4-oxadiazole (odt) derivatives and hydrazides (hz) were used as ligands for Pd(II) complexes, aiming to generate inhibitors of lipoxygenase (LOX) and butyrylcholinesterase (BChE). The Pd(II)-hz complexes (**P1**–**P3**) and six new Pd(II)-odt complexes (**P′2**–**P′7**), shown in Figure 36, were obtained via the reaction of the ligands with Pd(II) in a 1:2 metal/ligand molar ratio in acetonitrile or ethanol at room temperature [170].

All compounds synthesized exhibited moderate BChE inhibition with IC_50_ values ranging between 21.5 and 95.6 μM, which were lower than the IC_50_ of the reference drug eserine (IC_50_ = 7.3 µM). The most active complexes were **P1** and **P’2** with IC_50_ values of 21.5 and 23.4 µM, respectively [170].

## 5. Conclusions

Many diseases (infections, cancers, diabetes, parasitic diseases, Alzheimer’s disease, dementia, etc.) have concerned the scientific community due to the high mortality, high toxicity, and lack of effective drugs, as well as the presence of drug resistance to the available treatments. Aiming for new therapeutic solutions, the scientific community has synthesized the simplest to the most complex hydrazides to obtain new compounds with different biological activities. Most of the reported hydrazides in the last five years have been synthesized from acids or acid derivatives by reactions with hydrazine. A few new methods were also reported to synthesize hydrazides, such as the transamidation of N-substituted amides with hydrazine, the alkylation of hydrazides promoted by ruthenium catalysts, N-N cross-coupling reactions between hydroxamates and amines catalyzed by nickel complexes, nickel-catalyzed photochemical C-N coupling reactions between hetero(aryl) halides and hydrazides, photochemical reactions between azodicarboxylates and aryl or heteroaryl acylsilanes, and via hydrazine insertion into the β-position of benzoyl acrylates. The newly developed methods mostly generated substituted hydrazides, except for transamidation, which allowed the synthesis of non-substituted hydrazides in high yield. A wide variety of hydrazides have been used as synthons and precursors to hydrazide–hydrazones or heterocyclic derivatives. From hydrazides, hydrazide–hydrazone derivatives, oxadiazoles, and pyrazoles are among the most synthesized compounds. In addition, pyrrolones, thiazoles, thiadiazoles, and triazoles were also synthesized. Overall, the synthesis methods allow for obtaining these compounds in reasonable to excellent yields from hydrazides. The purification methods include silica gel column chromatography and recrystallization, with the latter being the most common. Hydrazides and their derivatives showed many biological activities, such as anticancer, antidiabetic (as alpha-glucosidase inhibitors), antibacterial, antifungal, antiparasitic, antiviral, anti-inflammatory (as COX inhibitors), and cholinesterase inhibition activity (neurodegenerative diseases), among others. This review represents a convenient tool for those aiming to explore the synthesis of new hydrazides or their derivatives to generate different scaffolds with biological activity.

## Figures and Tables

**Table 2:** Reaction conditions for the synthesis and purification of substituted hydrazides from acid or acyl chlorides.

Ref.	Starting Material	Experimental Conditions	Purification Process	Products (η%)
[106]	** 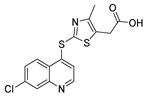 ** **30a**	1. CH_2_Cl_2_ EDC, DMAP, 0 °C, 1 h 2. RCONHNH_2_, r.t., 24 h	Recrystallization from ethyl acetate/CH_2_Cl_2_ (1:1)	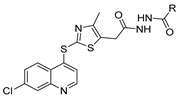 **29a** (72–88%)
[107]	** 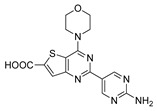 ** **30b**	RCONHNH_2_, EDC, DMF, r.t, 12 h	Silica column chromatography	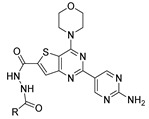 **29b** (68–78%)
[108]	** 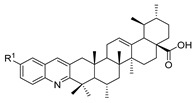 ** **30c**	1. SOCl_2_, benzene, reflux, 3 h 2. R^2^CONHNH_2_, Et_3_N, CH_2_Cl_2_/ether, r.t, 8–12 h	Silica column chromatography	** 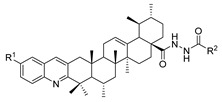 ** **29d** R^1^ = Halide, R^2^ = Alk (46–88%)
[109]	** 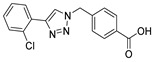 ** **30d**	RNHNH_2_, EDC, HOBT, Et_3_N, CH_2_Cl_2_, r.t., 12–24 h	Silica column chromatography	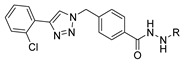 **28a** (35–97%)
[92]	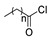 **19c**	**8aap**, CHCl_3,_ r.t., 1 h	-	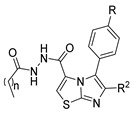 **29c** (65–70%)

n.s.—not specified by the authors.

**Table 3:** Reaction conditions for the synthesis and purification of hydrazide–hydrazones.

Ref.	Starting Material	Experimental Conditions	Purification Process	Hydrazone Compounds (η%)
[124]	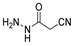 **8abd**	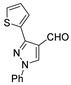 (1 eq) EtOH, reflux, 2 h	Recrystallization from ethanol	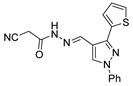 **52a** (71%)
[97]	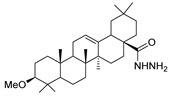 **8aaw**	RCHO (1.25 eq) MeCN:CHCl_3_ (1:1), HOAc (0.9 eq), 65 °C, 24 h R = Ar	Silica gel column chromatography	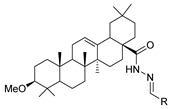 **52b** R = Ar (72–94%)
[68]	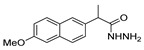 **8ah**	RCHO (1.1 eq) EtOH, glacial CH_3_COOH (drops), reflux, 8 h R = Ar	Recrystallization from ethanol	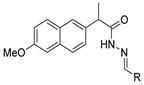 **52c** R = Ar (56–95%)
[96]	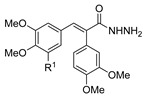 **8aau**	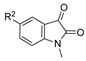 (1 eq) MeOH, CH_3_COOH (cat.), reflux, 1h	Flash column chromatography	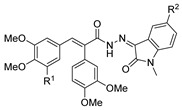 **52d** (63–85%)
[72]	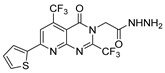 **8as**	RCHO (1 eq), EtOH, piperidine (0.3 eq), reflux, 2 h R = Ar	-	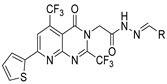 **52e** R = Ar (n.s.)
[77]	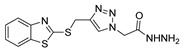 **8ao**	RCHO (1 eq), EtOH, CH_3_COOH (drops), reflux, 6–8 h R = Ar	Recrystallization from ethanol	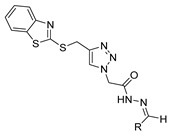 **52f** R = Ar (85–92%)
[57]	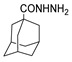 **8h**	RCHO (1 eq) EtOH, reflux R = Ar	-	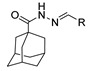 **52g** R = Ar (26–55%)
[57]	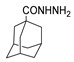 **8h**	MeCOR (1 eq) EtOH, reflux R = Ar	-	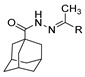 **52h** R = Ar (15–61%)
[75]	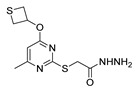 **8aq**	MeCOR^1^ (n.s.) EtOH, reflux, 1 h	Recrystallization from EtOH, n-BuOH, or *i*-PrOH	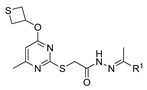 **52j** (61–89%)
[73]	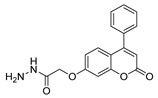 **8ap**	MeCOR (1 eq), CH_3_COOH (17 eq), EtOH, reflux, 6–8 h R = Ar	Recrystallization from acetic acid	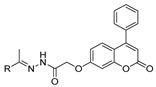 **52k** R = Ar (85–95%)
[101]	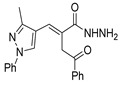 **8aba.1**	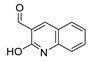 (1 eq) dioxane, reflux, 30 min	Recrystallization from ethanol/dioxane mixture (1:1) or dioxane	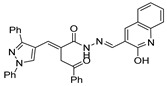 **52l** (77%)
[76]	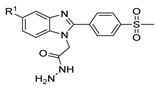 **8ar**	R^2^CHO (1 eq) EtOH, CH_3_COOH (cat.), reflux, 6 h R^2^ = Ar	Recrystallization from ethanol	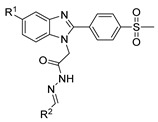 **52m** R^2^ = Ar (41–60%)
[76]	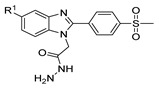 **8ar**	MeCOR (1 eq) EtOH, CH_3_COOH (cat.), reflux, 6 h R = Ar	Recrystallization from ethanol	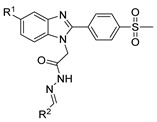 **52n** R = Ar (46–62%)
[117]	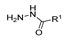 **8abe** R^1^ = H **8abf** R^1^ = CH_3_	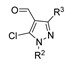 (1 eq) EtOH, CH_3_COOH (0.9 eq), r.t.	Recrystallization from dichloromethane/ethanol (1:2)	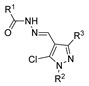 **52o** (40–84%)
[43]	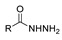 **8ai**–**8am**	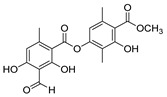 (antranorin) EtOH, CH_3_COOH, 50 °C, 2h	Silica gel column chromatography	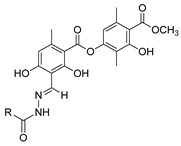 **52p** (73–89%) 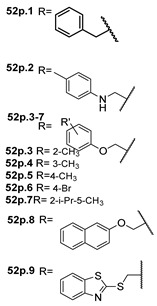
[55]	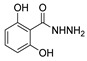 **8g**	RCHO (1 eq) MeOH, *p*-TsOH (cat.), r.t., 2 h R = Ar	Silica gel column chromatography	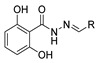 **52q** R = Ar (90–95%)
[56]	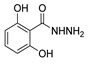 **8g**	RCHO (n.s.) MeOH, p-TsOH (cat.), r.t., overnight R = Ar	Silica gel column chromatography	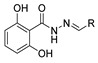 **52q** R = Ar (60–79%)
[95]	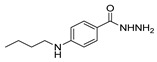 **8aat**	RCHO (1.1 eq) EtOH, CH_3_COOH (few drops), reflux, 12 h R = Ar	Recrystallization from ethanol	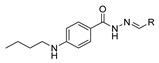 **52s** R = Ar (68–90%)
[66]	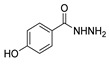 **8af**	RCHO (1.1 eq) EtOH, CH_3_COOH (few drops), reflux, 8 h R = Ar	Recrystallization from ethanol	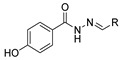 **52t** R = Ar (55–80%)
[67]	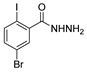 **8ag**	RCHO (1.1 eq) EtOH, reflux, 3 h R = Ar	Recrystallization from ethanol	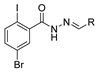 **52u** R = Ar (24–90%)
[118]	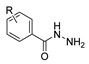 **8abj** R = 2-HO **8af** R = 4-HO	R^2^CHO (0.99 eq) EtOH or *i*-PrOH, 60 °C, 0.5–6 h R^2^ = Ar	Recrystallization from *i*-PrOH	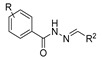 **52v** R = 2-HO, 4-HO; R^2^ = Ar (66–92%)
[38]	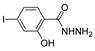 **8n**	RCHO (1.1 eq) EtOH, reflux, 3–35 min R = HetAr	Recrystallization from ethanol	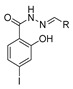 **52w** R = HetAr (18–97%)
[43]	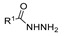 **8j**, **8k**	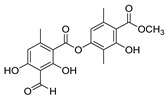 (n.s.) EtOH, CH_3_COOH, 50 °C, 2h	Silica gel column chromatography	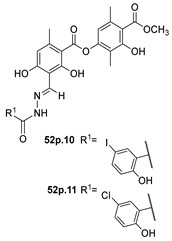 **52p** (73–89%)
[13]	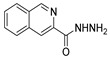 **8l**	2,4-(HO)_2_C_6_H_3_CHO (1 eq) MeOH, reflux, 6 h	-	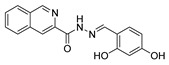 **52y** (84%)
[34]	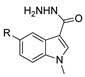 **8m**	1. 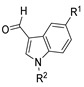 (1 eq) CH_3_COOH (28 eq), 90 °C, 6 h 2. NaHCO_3_	Recrystallization from ethanol	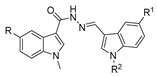 **52z** R, R^1^, R^2^ = alkyl or halide (86–90%)
[98]	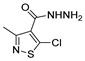 **8aax**	RCHO (1 eq), EtOH, 78 °C, 4 h R = Ar	Recrystallization from acetonitrile or 70% ethanol	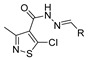 **52aa** R = Ar (50–86%)
[37]	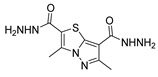 **8av**	RCHO (2.1 eq) EtOH/DMF, reflux, 5 h R = Ar	Recrystallization from DMF/ethanol	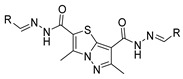 **52ab** R = Ar (72–80%)
[119]	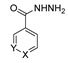 **1** X = N, Y = CH **8abg.1** X = CH, Y = N **8abg.2** X = N^+^-O^−^, Y = CH **8abg.3** X = CH, Y = N^+^-O^−^	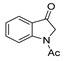 (n.s) EtOH or CH_3_COOH, reflux	n.s.	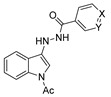 **52ac** (46–65%)
[119]	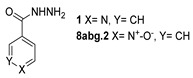	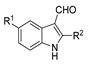 (n.s) EtOH or CH_3_COOH, reflux R^1^, R^2^ = alkyl	n.s.	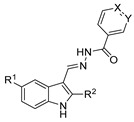 **52ad** R^1^, R^2^ = alkyl (72–95%)
[53]	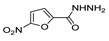 **8r**	RCHO (1.1 eq), EtOH, reflux, 2 h R = Ar	Recrystallization from ethanol	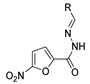 **52ae** R = Ar (10–93%)
[120]	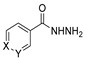 **1** X = N, Y = CH **8abg.1** X = CH, Y = N	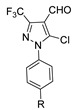 (1 eq) EtOH, reflux, 5 h	Silica gel column chromatography	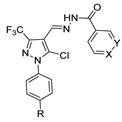 **52af** R = R = H, OCH_3_, F, Cl (89–94%)
[43]	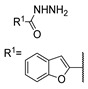 **8al**	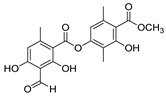 EtOH, CH_3_COOH, 50 °C, 2 h	Silica gel column chromatography	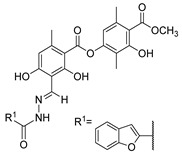 **52p.12**
[54]	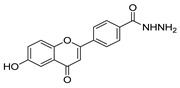 **8s**	R^1^COR^2^ (1 eq) EtOH, acetic acid (~0.2 eq), reflux, 8 h	-	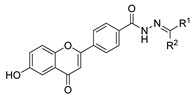 **52ag** R^1^ = Ar, R^2^ = H or alkyl (62–92%)
[121]	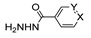 **1** X = N, Y = CH **8abh** X, Y = C-Br, C-Cl, CH, N, C-Me, C-NO_2_	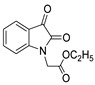 (1 eq) EtOH, CH_3_COOH (cat.), reflux, 3 h	Recrystallization from ethanol	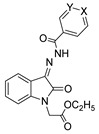 **52ah** (60–80%)
[122]	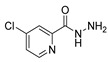 **8abi**	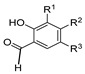 (1 eq) MeOH, reflux	Recrystallization from methanol	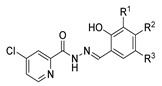 **52ai** (78–95%)
[123]	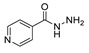 **1**	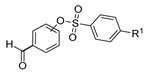 (1 eq) MeOH, CH_3_COOH (drops), reflux, 2 h	Recrystallization from ethanol	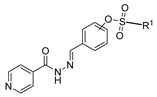 **52aj** R^1^ = Ar (75–87%)
[78]	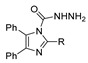 **8at**	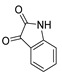 (n.s.), 25 min	-	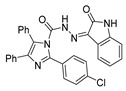 **52ak** R = Ar (93%)
[79]	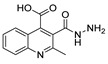 **8au**	4-HOC_6_H_4_CHO (1 eq) EtOH, CH_3_COOH (drops), r.t., 30 min	Recrystallization from ethanol	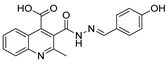 **52al** (23%)
[125]	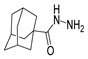 **8abh**	RCHO (1 eq), EtOH, reflux, 1–2h R = HetAr	Recrystallization from ethanol/water or ethanol	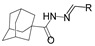 **52an** R = HetAr (82–95%)
[126]	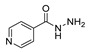 **1**	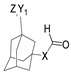 (1 eq), MeOH, Ar (g), reflux, 3–60 h; Y_1_: CHO, Z: H, Ph, cyclopentyl	Recrystallization from a mixture of chloroform/ether or silica gel column chromatography	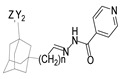 **52ao** (40–86%) Y_2_: CONH=CHPy; Z: H, Ph, cyclopentyl
[126]	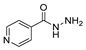 **1**	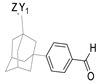 (1 eq), MeOH, Ar (g), r.t., 18–48 h Y_1_: CHO, Z: H, Ph, cyclopentyl	Recrystallization from a mixture of chloroform/ether or silica gel column chromatography	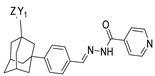 **52ap** (78–81%) Y_2_: CONH=CHPy; Z: H, Ph, cyclopentyl
[127]	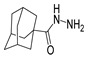 **8abh**	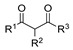 (1 eq), EtOH, CH_3_COOH (0.8 eq), reflux, 4–7 h R^1^ = Me or Ph; R^2^ = Me or H; R^3^ = Me or OC_2_H_5_	Recrystallization from ethanol	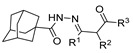 **52aq** (73%)

n.s.—not specified by the authors.

**Table 4:** Reaction conditions for the synthesis and purification of pyrrolones from hydrazides.

Ref.	Hydrazide	Experimental Conditions	Purification Process	Final Compounds (η%)
[27]	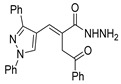 **8aba.2**	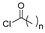 (n.s) Benzene, reflux	n.s.	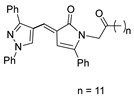 **53a** (78%)
[137]	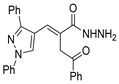 **8aba.2**	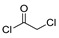 (1 eq, dropwise) DMF, r.t., 4 h	Recrystallization from ethanol	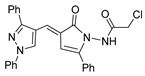 **53b** (78%)
[28]	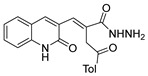 **8aax.1**	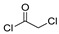 (1 eq) Et_3_N, dioxane, r.t., 1 h	Recrystallization from ethanol	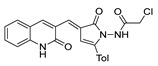 **53c** (86%)
[28]	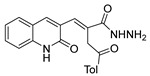 **8aax.1**	PhCOCl (1 eq) Benzene, reflux, 1 h	Recrystallization from ethanol	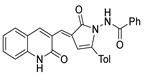 **53d** (68%)
[140]	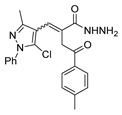 **8aax.2**	CH_3_COOCOCH_3_ (10.5 eq), r.t., 1 h	Recrystallization from benzene	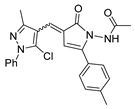 **53e** (65%)
[140]	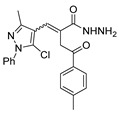 **8aax.2**	CH_3_COOCOCH_3_ (10.5 eq), reflux, 4 h	Recrystallization from petroleum ether (60–80)	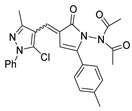 **53f** (60%)
[140]	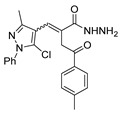 **8aax.2**	PhCOCl, benzene, reflux, 3 h	Recrystallization from benzene	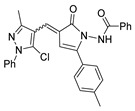 **53g** (60%)
[140]	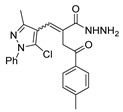 **8aax.2**	RCHO (1 eq), EtOH, CH_3_COOH, reflux, 3 h	Recrystallization from dioxane	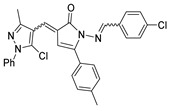 **54c** (80%)
[28]	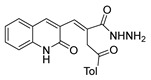 **8aax.1**	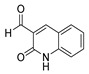 (1 eq), CH_3_COOH, ethanol, reflux, 1 h	Recrystallization from ethanol/dioxane mixture (1:1)	* 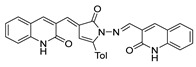 * **54a** (87%)
[101]	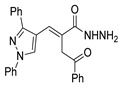 **8aba.2**	RCHO (1 eq) EtOH, CH_3_COOH (cat.), reflux, 1 h	Recrystallization from ethanol/dioxane mixture	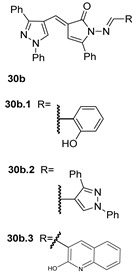 **54b** (80–84%)

n.s.—not specified by the authors.

**Table 5:** Reaction conditions for the synthesis and purification of pyrazoles, dihydropyrazoles, and pyrazolidine-dione from hydrazides.

Ref.	Starting Material	Experimental Conditions	Purification Process	Pyrazole Compounds (η%)
[80]	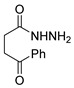 **8ac**	CH_2_(COOC_2_H_5_)_2_ C_2_H_5_ONa/EtOH Reflux, 14–17 h	Recrystallization from dioxane	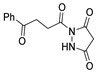 **71a** (90%)
[80]	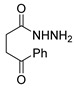 **8ac**	CH_3_COCH_2_COOC_2_H_5_ DMF K_2_CO_3_ Reflux, 12–15 h	Recrystallization from methanol	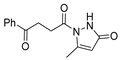 **69a** (85%)
[80]	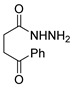 **8ac**	CNCH_2_COOEt DMF K_2_CO_3_ Reflux, 14–17 h	Recrystallization from methanol	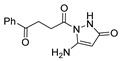 **68a** (77%)
[80]	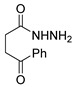 **8ac**	CNCH_2_COPh EtOH Reflux, 6–9 h	Recrystallization from dioxane	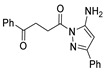 **65a** (72%)
[80]	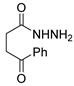 **8ac**	CH_3_COCH_2_COCH_3_ EtOH Piperidine (cat.) Reflux, 10–15 h	Recrystallization from methanol	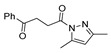 **64a** (95%)
[80]	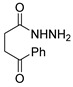 **8ac**	CNCH_2_CN EtOH Piperidine (cat.) Reflux, 20–25 h	Recrystallization from DMF	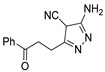 **66a** (70%)
[128]	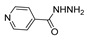 **1**	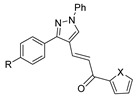 NaOH/EtOH r.t. 8–9 min	Recrystallization from methanol	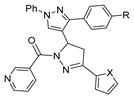 **67a** (82–88%)
[128]	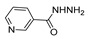 **8abg.1**	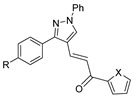 NaOH/EtOH r.t. 8–9 min	Recrystallization from methanol	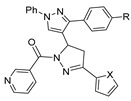 **67b** (82–87%)
[146]	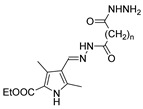 **8abk**	CH_3_COCH_2_COOC_2_H_5_ DMF/EtOH CH_3_COOH Reflux, 5 h	Column chromatography	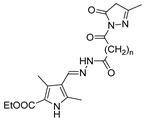 **70a** (62–67%)
[146]	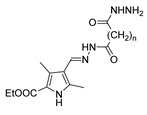 **8abk**	CH_3_COCH_2_COCH_3_ DMF/EtOH CH_3_COOH Reflux 8 h	Column chromatography	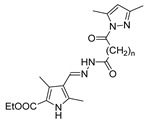 **64b** (61–72%)

**Table 6:** Reaction conditions for the synthesis and purification of 1,3,4-oxadiazoles and their derivatives.

Ref.	Starting Material	Experimental Conditions	Purification Process	Oxadiazole Compounds (n%)
[154]	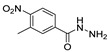 **8abl**	1. RCHO EtOH, reflux, 3 h 2. Ac_2_O (32 eq), reflux, 3 h	Recrystallization from ethanol/acetone (3:1 *v*/*v*) mixture	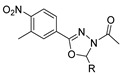 **78a** R = Ar (22–76%)
[82]	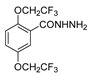 **8ax**	1. RCOR^1^ 70 °C, 2 h 2. Ac_2_O (10.5 eq), reflux, 8 h	Recrystallization from ethanol	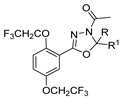 **78b** R = Ar, R^1^ = H, CH_3_ (n.s.)
[40]	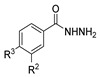 **8o** R^2^ = H, R^3^=H **8p** R^2^ = Cl, R^3^ = H **8q** R^2^ = H, R^3^ = Cl	1. 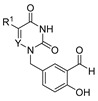 EtOH, CH_3_COOH (cat.) 80 °C, 3 h 2. Ac_2_O (10.5 eq), 155 °C, 90 min	Flash chromatography	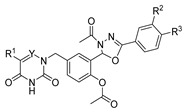 **78c** R^1^ = CH_3_, H, F; Y = CH, N; R^2^ and R^3^ = H, Cl (40–71%)
[153]	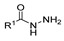 **8** R^1^ = Alk, Ar	1. R^2^CHO, t-BuOH, reflux, 2–3 h 2. NaOCl, r.t., 1–2 h	Column chromatography	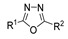 **77** R^1^ = Alk, Ar; R^2^ = Alk, Ar (50–93%)
[80]	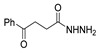 **8ac**	Ac_2_O, 10–15 min, EtONa, 6–8 h;	Recrystallization from ethanol	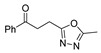 **77a** (88%)
[80]	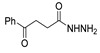 **8ac**	Ac_2_O, reflux, 25–30 h	Recrystallization from methanol	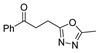 **77b**
[84]	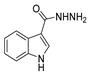 **8aab**	R-X-CO_2_H (1 eq) POCl_3_ (5 eq), 50 °C, 4 h X-R = CH_2_O-Ar, CH_2_-Ar or Ar	Recrystallization from ethanol	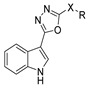 **77c** X-R = CH_2_O-Ar, CH_2_-Ar or Ar (46–66%)
[152]	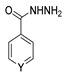 **1** Y = N or **8o** Y = CH	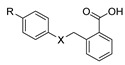 (1 eq) POCl_3_ (48 eq), reflux, 9 h	Recrystallization from an appropriate solvent	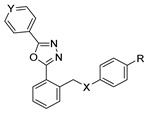 **77d** X = S, SO_2_; R = H, CH_3_, Cl; Y = N, CH (59–70%)
[83]	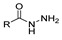 **8o** R = C_6_H_5_ **8ay** R = 4-F-C_6_H_4_ **8az** R = 4-CH_3_–C_6_H_4_ **8aaa** R = 2-Cl,4-Cl-C_6_H_3_	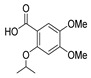 (1 eq), POCl_3_ (n.s.), reflux 6–7 h	Recrystallization from isopropanol	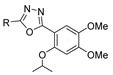 **77e** R = Ar (80–88%) **77e.1** R = C_6_H_5_ **77e.2** R = 4-F-C_6_H_4_ **77e.3** R = 4-CH_3_–C_6_H_4_ **77e.4** R = 2-Cl,4-Cl-C_6_H_3_
[2]	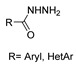 **8**	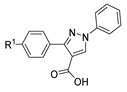 R^1^ = H, CH_3_, F (1 eq), POCl_3_ (53 eq) 60–70 °C	Recrystallization from methanol	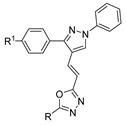 **77f** R = Aryl, HetAr; R^1^ = H, CH_3_, F (80–89%)
[59]	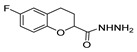 **8u**	R^1^CO_2_H (1 eq), POCl_3_ (6.7 eq), reflux, 6–8 h	Recrystallization from methanol	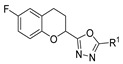 **77g** R^1^ = Ar (78–90%)
[105]	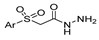 **8v**	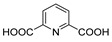 (0.5 eq) POCl_3_ ultrasonication (35 kHz) 35–60 min	Recrystallization from 2-propanol	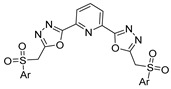 **77h** (80–92%)
[105]	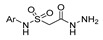 **8w**	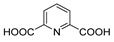 (0.5 eq) POCl_3_ (53 eq) ultrasonication (35 kHz) 35–50 min	Recrystallization from 2-propanol	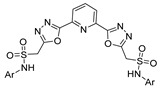 **77i** (87–91%)
[81]	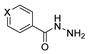 **1** X = N or **8o** X = CH	1. CH_2_Cl_2,_ 0 °C ClCOCOOEt (1.1 eq) Et_3_N r.t., 8 h 2*. p*-TosCl (1 eq) r.t., 4 h	Recrystallization from ethanol	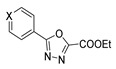 **79a** X = N or CH (87, 83%)
[39]	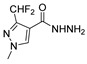 **8t**	ClCOCOOCH_3_ (1.1 eq) POCl_3_ (10 eq), 85 °C 4–6 h	Recrystallization from ethanol	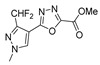 **79b** (n.s.)
[86]	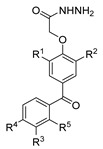 **8aaj**	CS_2_ (17 eq) KOH (2 eq) EtOH, reflux, n.s. time HCl (pH ~3–4)	Recrystallization from acetone	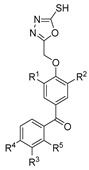 **80a** R^1^ = H, CH_3_, F R^2^ = H, Cl R^3^ = H, Cl R^4^ = H, Cl, I R^5^ = H, Cl (84% as an example)
[64]	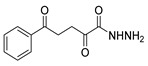 **8ac**	1. CS_2_ (5 eq) KOH (1.5 eq), reflux, 3 h 2. HCl (pH ~4)	Recrystallization from EtOH/DMF or EtOH	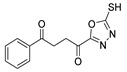 **80b** (62%)
[36]	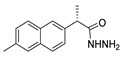 **8z**	1. CS_2_ (n.s) KOH (n.s.) EtOH, r.t., 24 h 2. 90–100 °C, 10 h	n.s.	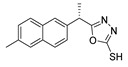 **80c** (n.s)
[37]	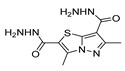 **8v**	CS_2_ (2.5 eq), KOH (2 eq) EtOH, reflux, 3h 2. HCl	Recrystallization from ethanol/DMF	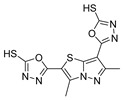 **80d** (40%)
[146]	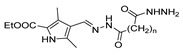 **8abk**	KOH (2 eq), CS_2_ (2 eq) DMF, HCl (cat.), reflux, 30 min	Column chromatography	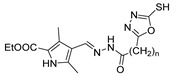 **80e** (52–57%)
[63]	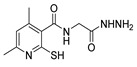 **8abm**	1. CS_2_ (4 eq) KOH (2 eq) EtOH, reflux, 10 h 2. HCl	Recrystallization from ethanol	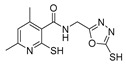 **80f** (65%)
[41]	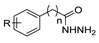 **8aa**	1. CS_2_ (1.5 eq)_,_ KOH (1.5 eq), EtOH, r.t., 72 h 2. HCl	-	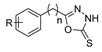 **80g** (64–70%)
[85]	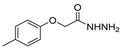 **8al**	1. CS_2_ (2 eq), KOH (1 eq), EtOH, reflux, 4–5 h 2. HCl (pH ~5–6)	-	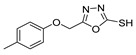 **80h** (95%)
[61]	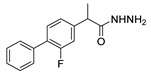 **8x**	1. CS_2_ (2 eq) KOH (0.5 eq) EtOH, reflux, 12 h 2. HCl	Recrystallization from ethanol	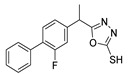 **80i** (74%)
[87]	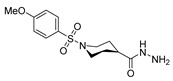 **8aak**	1. CS_2_ (1.5 eq) KOH (1 eq) MeOH, reflux, 2 h 2. HCl (pH 4–5)	-	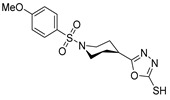 **80j** (n.s.)
[62]	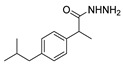 **8y**	1. CS_2_ (2 eq) KOH (1.5 eq) EtOH, reflux 80 °C, 6–8 h 2. HCl (pH 2–3)	Recrystallization from ethanol	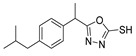 **80k** (83%)
[28]	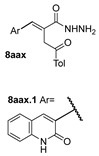	CS_2_ (1.1 eq), pyridine, 70–75 °C, 4 h	Recrystallization from ethanol	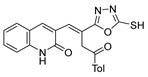 **80l** (63%)
[103]	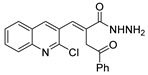 **8abb.1**	CS_2_ (1.1 eq), NaOH, reflux, 12 h	Recrystallization from a benzene/ethanol mixture (2:1)	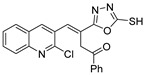 **80m** (n.s.)
[86]	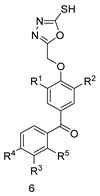 **80a**	ClR.HCl (3 eq), K_2_CO_3_, DMF, reflux, 10–15 h	-	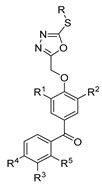 **81a** R = Alk (70–89%)
[64]	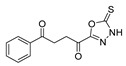 **80b**	C_2_H_5_I, NaOH/EtOH, reflux, 6 h	-	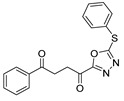 **81b** (60%)
[64]	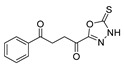 **80b**	PhCOCH_2_Br, acetone, reflux, 6 h	-	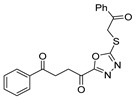 **81c** (50%)
[36]	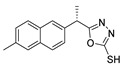 **80c**	1. 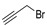 , K_2_CO_3_, acetone, 50–60 °C, 4 h 2. RN_3_, H_2_O-^t^BuOH (1:1), CuSO_4_·5H_2_O, C_6_H_7_O_6_·Na, r.t., 4–12 h	-	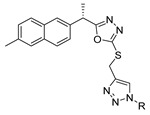 **81d** R = Ar (n.s)
[85]	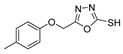 **80h**	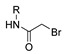 KOH, EtOH, r.t., 4–5 h	-	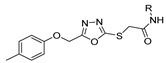 **81e** R = Alk, Ar (92–98%)
[61]	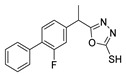 **80i**	RCH_2_Cl (1 eq), KOH (1 eq), H_2_O, acetone	-	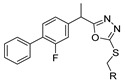 **81f** R = Alk, Ar (67–76%)
[87]	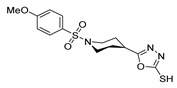 **80j**	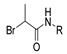 (1 eq) LiH, DMF, r.t. or with microwave irradiation	-	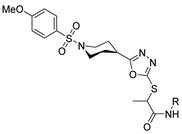 **81g** R = Ar (84–96%)
[62]	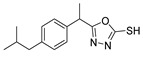 **80k**	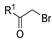 K_2_CO_3_, EtOH, reflux, 5–6 h	-	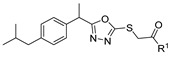 **81h** R^1^ = Ar (58–79%)

n.s.—not specified by the author.

**Table 7:** Reaction conditions for the synthesis and purification of 1,3-thiazole and 1,3,4-thiadiazole derivatives from hydrazides.

Ref.	Starting Material	Experimental Conditions	Purification Process	Products (η%)
[64]	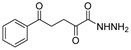 **8ac**	CS_2_, KOH (aq.), reflux, 3 h	-	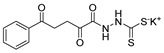 **86a** (86%)
[64]	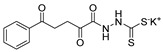 **86a**	PhCOCH_2_Br, EtOH, reflux, 3 h	Recrystallization from ethanol	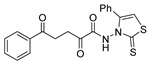 **89a** (67%)
[44]	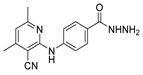 **8aaq**	PhNCS, EtOH, reflux, 4 h	-	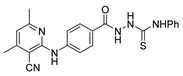 **87a** (64%)
[44]	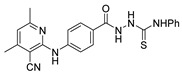 **87a**	ClCH_2_COOH, EtOH, AcONa, reflux, 6 h	-	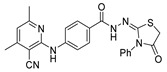 **90a** (74%)
[108]	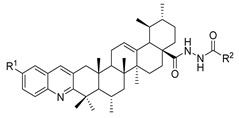 **29d**	Lawesson reagent (1.7 eq) toluene, 110 °C, 6 h	Silica gel column chromatography	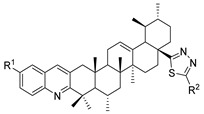 **92a** (36–49%)
[93]	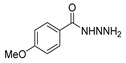 **8aar**	1. Lawesson’s reagent (1 eq) toluene, reflux, 8 h 2. RCHO (1 eq), POCl_3_ (107 eq) reflux, 4–6 h	Recrystallization from methanol	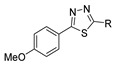 **92b** (n.s)
[94]	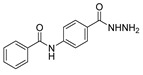 **8aas**	1. R^1^NCS, EtOH, 2 h 2. H_2_SO_4_, reflux, 5 h	1. Recrystallization from ethanol 2. Recrystallization from ethanol	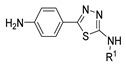 **91a** (n.s.)

n.s.—not specified by the authors.

**Table 8:** Reaction conditions for the synthesis and purification of 1,2,4-triazole derivatives from hydrazides and their derivatives.

Ref.	Starting Material	Experimental Conditions	Purification Process	Intermediates or Triazoles (η%)
[35]	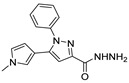 **8ad**	NH_4_SCN (1 eq) HCl, EtOH, reflux, 6 h	Recrystallization from DMF/ethanol	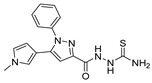 **87a** (82%)
[35]	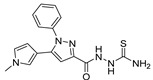 **87a**	KOH EtOH, reflux, 6 h	Recrystallization from DMF/ethanol	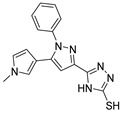 **97a** (78%)
[80]	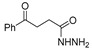 **8ac**	KSCN (1 eq), 10% HCl, reflux, 6–9 h	Recrystallization from dioxane	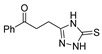 **97b** (77%)
[42]	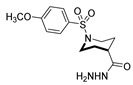 **8aal**	1. MeNCS (1 eq) EtOH, reflux, 1 h 2. KOH (1 eq), reflux, 1 h 3. HCl	-	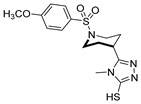 **97c** (n.s.)
[42]	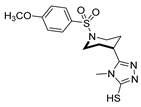 **97c**	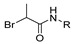 (1 eq) LiH, DMF, r.t, 6–17 h or LiH, DMF, microwave, 33–90 s	-	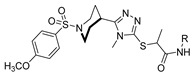 **100a** R = Ar (85–90%)
[88]	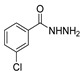 **8p**	EtNCS (1 eq) EtOH, reflux 3–4 h	-	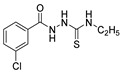 **87d** (98%)
[88]	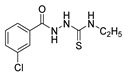 **87d**	10% NaOH, reflux, 4 h HCl	-	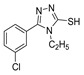 **97d** (96%)
[88]	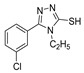 **97d**	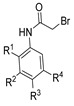 (1 eq) DMF, NaH, 30 min, r.t.		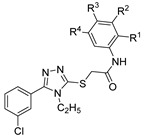 **100b** (62–77%)
[89]	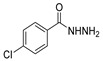 **8q**	furfuryl isothiocyanate (1 eq) MeOH, reflux, 8–10 h	-	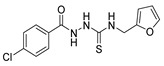 **87e** (97%)
[89]	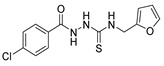 **87e**	10% NaOH reflux, 6–8 h HCl	-	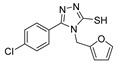 **97e** (94%)
[89]	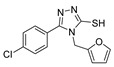 **97e**	EtOH, KOH(1 eq) 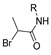 (1 eq), reflux, 4–5 h	-	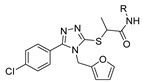 **100c** R = Ar (89–95%)
[90]	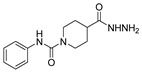 **8aao**	EtNCS or PhNCS (1 eq) EtOH, reflux 3–4 h	-	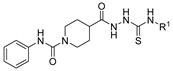 **87f** (97%)
[90]	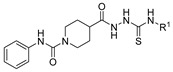 **87f**	1. 10% NaOH, reflux 4 h 2. HCl	-	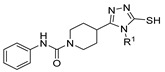 **97g** (98%)
[90]	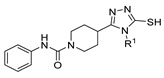 **97g**	KOH, EtOH, r.t., 30 min, alkyl halides (1 eq), reflux, 4–5 h	-	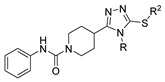 **100d** R^1^ = Ar, R^2^ = Alk (70–99%)
[91]	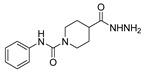 **8aao**	PhNCS MeOH, reflux 3–4 h	-	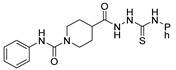 **87h** (98%)
[91]	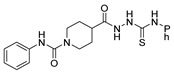 **87h**	1.10% NaOH, reflux, 4 h 2. HCl	-	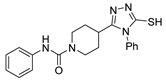 **97h** (95%)
[91]	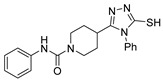 **97h**	KOH MeOH, r.t., 30 min 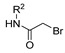 , reflux 4–5 h	-	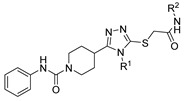 **100e** R^1^ = Ar, R^2^ = Ar (88–96%)
[165]	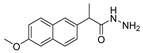 **8abn**	4-FC_6_H_4_NCS (1 eq), CH_3_(CH_2_)_3_OH, 65 °C, 4 h	Recrystallization from ethanol	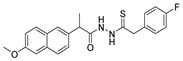 **87h** (75%)
[165]	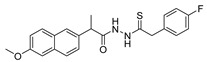 **87h**	4 N NaOH, heat under Radley, 16 h, CH_3_COOH	Recrystallization from ethanol	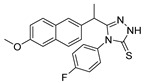 **97i** (60%)
[64]	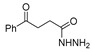 **8ac**	CS_2_ KOH (aq.), reflux, 3 h	-	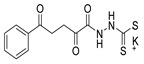 **86a** (86%)
[64]	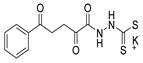 **86a**	H_2_NNH_2_·H_2_O, reflux 2 h	Recrystallization from EtOH/DMF	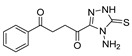 **98a** (43%)
[64]	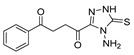 **98a**	PhCOCH_2_Br (1 eq), Et_3_N (1 eq), EtOH, reflux, 2 h	Recrystallization from DMF/H_2_O	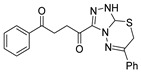 **101** (60%)
[41]	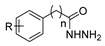 **8aa**	KOH CS_2_ water/ethanol (1:1) H_2_NNH_2_·H_2_O 64% (1 eq), reflux	-	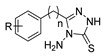 **98b** (52–65%)
[41]	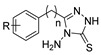 **98b**	RCHO (1 eq), CH_3_COOH (cat.), MeOH, reflux, 12 h	-	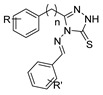 **102** (72–89%)
[166]	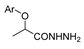 **8abo**	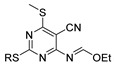 (1 eq), 2-methoxyethanol TFA (cat.) reflux, 8–10 h	Recrystallization from DMSO	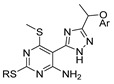 **99a** (70–84%)
[167]	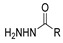 **8** R = Ar	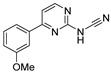 (1 eq) DMF, 90 °C, 5 h HCl (0.05 N)	Recrystallization from dioxane	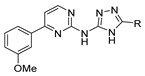 **99b** R = Ar (73–81%)

n.s.—not specified by the authors.

## Data Availability

No data was used for the research described in the article. Data sharing is not applicable.
